# Supplementary figures and images for: Cervical cancer screening rates before and after the Great East Japan Earthquake in the Miyagi Prefecture, Japan
Source: PLoS One. 2020 Mar 11;15(3):e0229924. doi: 10.1371/journal.pone.0229924 (PMC7065810; doi:10.1371/journal.pone.0229924)

## Slide 1
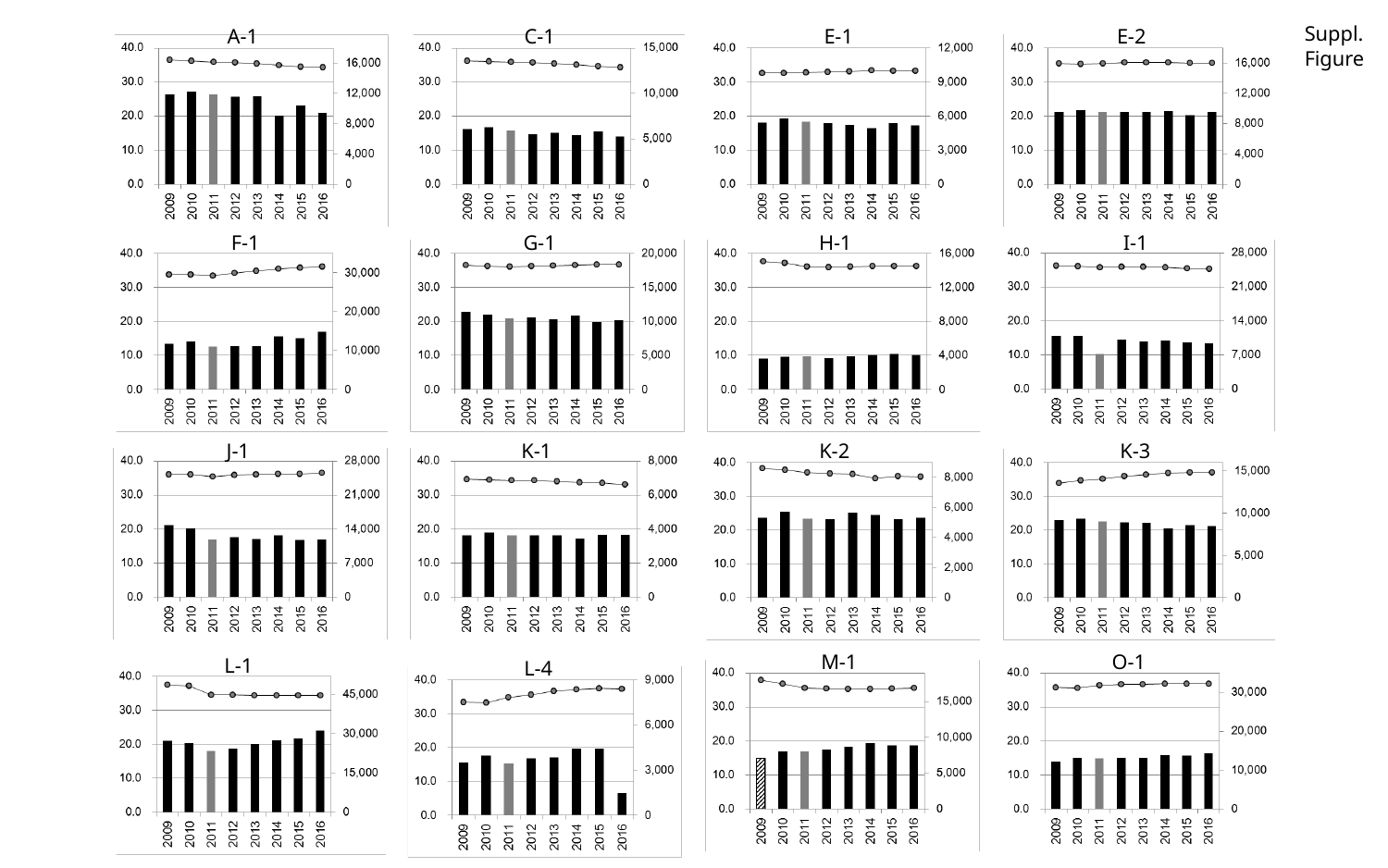

Suppl.
Figure
A-1
C-1
E-1
E-2
F-1
G-1
H-1
I-1
J-1
K-1
K-2
K-3
M-1
O-1
L-1
L-4

## Slide 2
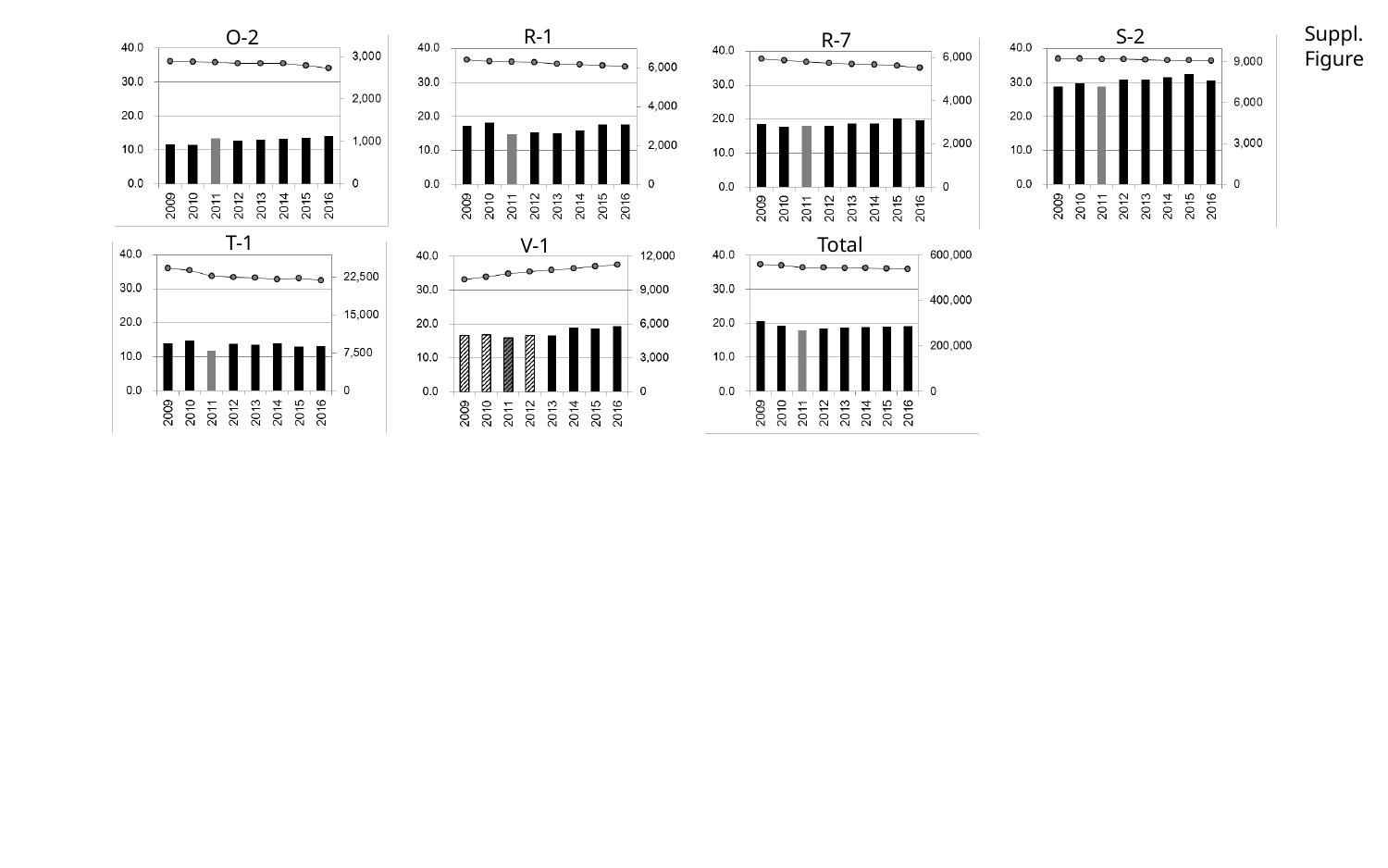

Suppl.
Figure
R-1
S-2
O-2
R-7
T-1
Total
V-1

Supplement: S1 Fig — The bar graph shows the CCS-R (left vertical axis; unit, %), and the line graph shows the population (right vertical axis; unit, number of people). (PPTX) [file pone.0229924.s002.pptx]
