# Supplementary material for: Cervical cancer screening rates before and after the Great East Japan Earthquake in the Miyagi Prefecture, Japan
Source: PLoS One. 2020 Mar 11;15(3):e0229924. doi: 10.1371/journal.pone.0229924 (PMC7065810; doi:10.1371/journal.pone.0229924)
Supplement: Attachment — Submitted filename: PONE-D-19-25407_R1 (1).pdf [file pone.0229924.s004.pdf]

PLOS ONE

Cervical cancer screening rates before and after the Great East Japan Earthquake in Miyagi Prefecture, Japan

--Manuscript Draft--

**Manuscript Number:**	PONE-D-19-25407R1
**Article Type:**	Research Article
**Full Title:**	Cervical cancer screening rates before and after the Great East Japan Earthquake in Miyagi Prefecture, Japan
**Short Title:**	Cervical cancer screening and the Great East Japan Earthquake
**Corresponding Author:**	Kiyoshi Ito<br>Tohoku University<br>Sendai, JAPAN
**Keywords:**	cervical cancer screening; The Great East Japan Earthquake; disaster medicine; Women's Health
**Abstract:**	After disasters, issues pertaining to women's health such as irregular periods and bleeding are well surveyed. However, the management of women's health, especially changes in the rate of health checkups, has not been investigated. In the present study, we focused on the change in the cervical cancer screening rates (CCS-Rs) before and after the Great East Japan Earthquake in Miyagi Prefecture, Japan. The earthquake had a magnitude of 9.0, a profound disaster. We examined the CCS-R from 2009 to 2016 in 45 areas of the Miyagi Prefecture. Screening was completed using mobile vans. In the 4 areas impacted by the tsunami after the earthquake, a marked decrease in the CCS-R was observed in 2011 when the earthquake took place (more than a 3% decrease compared with that in the previous year). The CCS-Rs in these 4 regions remained lower in 2016 than in the previous year. In 2009–2016 except for 2014, CCS-Rs in coastal areas (9 areas) were significantly lower than those in the non-coastal areas (36 areas). A delay in seeking healthcare, also known as "patient's delay," is considered as one of the problems of cancer treatment in affected areas. It is possible that a decrease in the CCS-R may lead to low detection of advanced stages of cancer. Therefore, the establishment of a comprehensive medical system including medical screening after a disaster is important for the management of women's health.
**Order of Authors:**	Yasuhiro Miki
	Toru Tase
	Hideki Tokunaga
	Nobuo Yaegashi
	Kiyoshi Ito
**Response to Reviewers:**	To Reviewer #1:<br>We appreciate your comments regarding our manuscript entitled "Cervical cancer screening rates before and after the Great East Japan Earthquake in Miyagi prefecture, Japan" (manuscript ID: PONE-D-19-25407). We have revised the manuscript according to your comments and provided detailed responses to the specific comments below.<br><br>Maybe have an editor copy edit for grammar.<br>This manuscript has been strictly edited.<br><br>Abstract: say earthquake was a magnitude 9.0 to show the seriousness of the disaster.<br>We have added the above to the abstract accordingly (lines 22-23).<br><br>Intro: authors say screening rates are low in Japan compared to other countries. What are the prevalence rates of cervical cancer in Japan compared to other countries? What about HPV vaccine rates?<br>We appreciate your comment. We believe it is important to provide information on

Powered by Editorial Manager® and ProduXion Manager® from Aries Systems Corporation

cervical cancer prevalence and vaccination rates in Japan. We have summarized that information in the Introduction section (lines 36-44; 46-47; 49-50).

Why was it called the 3.11 earthquake?
The abbreviation "3.11" has been used mainly by Japanese mass media since the earthquake occurred on March 11, 2011. We described the above in the Introduction section (lines 57-59). In addition, “3.11” is widely recognized in Japan; however, there may be many readers who are not familiar with this term. Therefore, we have not used “3.11” as an abbreviation in the manuscript.

What is the evidence to support this line: However, the people affected by the disaster are 61 still unstable, and cannot afford to take care of their health.
Several studies have reported that the mental and financial instability of survivors creates “patients’ delay” (lines 70-71). We have added references to clarify this and described it in the next paragraph in the revised manuscript (lines 72-82).

Why study cervical cancer and not the other women’s health conditions? Not well justified
We have summarized this information in the Introduction and have described it in the Discussion section. We clarified why we targeted a disaster and its impact on cervical cancer screening in this research (lines 72-86).

Table 1 is not useful to a reader unfamiliar with the region. A map would be more useful.
The code numbers in Table 1, which is S1 Table in the revised version, match those in Figure 1B in order to distinguish the areas on the map. It is clearly described in each legend in the S1 Table and Figure 1B in the revised manuscript.

Methods: Is the Miyagi Cancer Society a reputable resource? How do they get their data? Is it a government agency?
The Miyagi Cancer Society was the first established organization in Japan for cancer since 1958. In 2012, the society was recognized by the administrative agency as a public interest incorporated foundation and has undertaken a cancer screening and cancer registration business in Miyagi Prefecture, Japan. The Annual Report is also compiled as one of its functions. We have summarized these details in the Materials and Methods (lines 100-103).

Results: Why only mobile van data included? Seems like a significant limitation. Where do most people get cervical cancer screenings in this region?
Medical exam statics at hospitals or the medical center in Miyagi Prefecture include a large number of occasional outpatients, who complain of symptoms such as irregular bleeding. On the other hand, women who have undergone cervical cancer screening in a mobile van were all periodic health care check-ups. In addition, in these regions, cervical cancer screening is covered by the mobile van. We added above sentences in the Materials and Methods and Results (lines 117-122).

Better visualizations would make the data more meaningful (a map rather than a long table, perhaps). AH OK, I see maps at the end. I like the figures. But better to label the map with the region name and the bar charts with region names. The “T-3” labels are hard to follow.
We agree with your comment. We revised Figure 1B accordingly.

Discussion: what were the results from the pregnancy studies? I still don’t know why cervical cancer was the focus here. No solid health or financial justification has been provided. OK page 19-21 are good. Maybe more of this justification or background should be in the introduction.
We agree with your suggestion. We have included a summary of this discussion in the introduction and have clarified our motivations for studying a disaster and its impact on cervical cancer screening (lines 72-86).

What was the population decline?
It is well known that the tsunami resulted in severe human and residential damage as a result of the Great East Japan Earthquake. Therefore, coastal populations are considered to have decreased both due to death as well as migration as a

Powered by Editorial Manager® and ProduXion Manager® from Aries Systems Corporation

consequence of the tsunami. We added this sentence in the Discussion section (lines 227-230).

Overall it’s a decent paper. Though I am still unclear if the magnitude of change in screening is clinically meaningful. Can that point be proven? For example, saying that in Katrina the mean time to diagnosis was 7 years compared to 4 before Katrina – that is clinically meaningful. Could something similar be said here?

We appreciate your kind comments regarding our research. Compared to the USA CCS-R, the Japan CCS-R is too low. Therefore, it would be difficult to perform the same analysis as that in US research due to the small number of comparative patients. Otherwise, in Japan, cervical cancer screening is expected to reduce cervical cancer deaths (recommendation grade B), with evidence levels of 2++ for conventional and 2+ for liquid-based cytology. There are limitations to conducting a further detailed analysis in the areas examined in this study; however, a reduction in CCS-Rs suggests that women’s health will be significantly affected. We have clarified this aspect in the Discussion section (lines 274-286).

To Reviewer #2
We appreciate your comments regarding our manuscript entitled “Cervical cancer screening rates before and after the Great East Japan Earthquake in Miyagi prefecture, Japan” (manuscript ID: PONE-D-19-25407). We have revised the manuscript according to your comments and provided detailed responses to specific comments below.

It may be more universally understandable if the magnitude of the earthquake is given in the abstract, i.e., after the Great East Japan Earthquake to put in the parentheses (magnitude 11.0); if the authors decide to leave 3.11 earthquake in the parentheses, they should put it in quotations "3.11" or indicate in some other way that this is a colloquial, synonymous term. The authors do explain this in line 53, however (but the abstract is often the first paragraph read in a manuscript). Elsewhere in the manuscript (line 53, 59), it may be more clear if the authors refer to the earthquake as the Great East Japan Earthquake instead of the 3.11 earthquake.
We agree with your suggestions. In the abstract, “3.11” has not been used as an abbreviation for the Great East Japan Earthquake. In the text, “3.11” was only mentioned as being used mainly by the Japanese mass media and not used as an abbreviation elsewhere (lines 57-59).

In the abstract, the sentence "It is possible that a decrease in CCS-R will lead to the detection in the advanced stages of cancer." may be incorrect? Did the authors want to suggest that a decrease in CCS-R may lead to less detection of advanced stages of cancer?
We apologize for any inaccuracies. As you pointed out, a decrease in the CCS-R may lead to low detection of advanced stages of cancer. We have revised the abstract accordingly (lines 31-32).

In the first sentence of the introduction (line 36), please state why this recommendation is important (otherwise this sentence seems abrupt as an opening to this manuscript).
We agree with your comments. It is important to consider that in the results of this study, cervical cancer screening is recommended based on the evidence from Japan. Therefore, we moved this sentence from the Introduction to the Discussion section in the revised manuscript.

The introduction does not flow well in terms of the verbage used. Lines 41-45 should flow and lead into each other.
We agree with your comments. We have revised this series of sentences in the revised manuscript (lines 36-55).

Lines 55-57 use past and present tense terms. Instead of "may need 10 years for reconstruction" perhaps the authors may consider using "the tsunami-hit area was predicted to necessitate 10 years for reconstruction."
We agree with your comments. We have revised this sentence accordingly (lines 62-63).

Powered by Editorial Manager® and ProduXion Manager® from Aries Systems Corporation

	Please give a time course in lines 57-61. How long after the earthquake did the mobile van service resume screening?<br>We agree with your comments. In many areas, cervical cancer screening was resumed in April of the year of the disaster . However, in coastal areas (L-2, L-3, T-3, U-1b in Fig. 1), the re-initiation of screenings was delayed from July to December of that same year . The screening was resumed only in Oshika (L-7 in Fig. 1) in February following the earthquake. We added this information to the Introduction section (lines 66-70).<br><br>Line 62: please cite this publication within the manuscript using a standardized method such as MLA format (i.e., "In a study describing survey results regarding reproductive health after the 2008 Wenchuan earthquake in China, Liu et al. reported......")<br>We agree with your comments. In the revised manuscript, we modified the Introduction section to include this sentence (lines 72-86).<br><br>Lines 69-71 is too nebulous and general of a statement to make. The authors should add in that the failure of health management in the specific context of natural disasters (or whatever they feel it is specifically pertaining to).<br>We agree with your comments. In the revised manuscript, we modified the Introduction section accordingly (lines 72-86).<br><br>Line 72: "Japan fiscal year (FY; April 1 to March 31) 2009–FY2016" should be simplified to set years (i.e., 2009-2016).<br>We agree with your comments. We have revised the manuscript accordingly (line 87).<br><br>Table 1 is too complex and slightly unnecessary. Perhaps a small color coded index included as a legend in Figure 1 with the names of the prefectures would suffice (with consideration to take out Table 1 entirely; the prefectures can remain in Table 2 since it also provides pertinent information).<br>We agree that Table 1 may be unnecessary for many readers. However, it may be helpful for readers who know Japan in order to identify a location. We have left it as a supplemental table. In addition, we have revised Figure 1 to make it easier to understand.<br><br>Line 93: instead of "people," should this be "females"? Why is the CCS-R pertaining to those 20 years old and over - if this is a screening protocol in Japan then please state that here.<br>We appreciate your polite comment. Line 93 "people" has been corrected to "women". The incidence of cervical cancer has increased in Japanese women in their mid-20s. Therefore, Japanese cervical cancer screening guidelines recommend to begin screening at the age of 20 years. We described this in the Materials and Methods section (lines 105-107).<br><br>It would be useful to get an idea about the numbers affected since -3.0% etc...(e.g., line 113, line 121) may be difficult to interpret meaningfully to the reader. The authors should consider detailing the population numbers in the text and delineating the axes on the graphs in all of the figures themselves (including the supplementary ones) for whatever they represent (i.e., year, population in thousands etc...) instead of in the figure legend.<br>We agree with your comments. We have added new graphs in Figures 2 and 3 to make it easier to understand the decrease in cervical cancer screening rates. We also revised the graphs accordingly.<br><br>The authors may consider not stating: "no significant difference was found" in line 170 since the p value was 0.6303. Likewise, in line 180 it is stated that there was a significant decrease in the coastal area compared with that in other areas however this is difficult to interpret without a regression analysis (and consideration of determining a p value).<br>We apologize for failing to explain Figure 5. We divided the regions into coastal and non-coastal areas and compared them using the Mann–Whitney U test. The p-value shows the results. We have added the legend for Figure 5 (lines 211-215)..<br><br>Lines 196-200 are unclear in their message?<br>In this article, it was suggested that the victims may be motivated to manage their health if they realize that there is recovery from disaster. However, there is no evidence

Powered by Editorial Manager® and ProduXion Manager® from Aries Systems Corporation

	or reference to support this suggestion as you point out. I agree that this is unclear. Therefore, we have deleted this text from the revised manuscript.<br><br>Lines 204-206: even though the authors could not identify causes, are there any that could be hypothesized based on data from previous similar studies?<br>We agree with your comments. In an evidence review by CPSTF (USA), small media sources such as newspapers and educational videos as well as solicitation by telephone or letter are recommended as interventions to increase the CCS-R (https://www.thecommunityguide.org/content/task-force-findings-cancer-prevention-and-control; access 2020.1.17). In Japan, it was also reported that local government initiatives and education contributed to increasing the screening rate of young women. However, a detailed investigation is required in areas where these screening rates have declined and awareness attempts by local government and other organizations may have been delayed. We have added this to the Discussion section (lines 248-257).<br><br>I was not able to easily find the information for reference #23. Is New Orlando, USA a city that was described in this study?<br>Ref.#23 is an abstract of the 2001 Western Association of Gynecologic Oncologists (WAGO) Annual Meeting published in Gynecologic Oncology. We noted this in the manuscript that this reference is a conference abstract. The DOI number is also added to link to this abstract (lines 78-80).<br><br>Line 218: what stage is being referred to? Lines 219-221 should be considered being combined.<br>The details of the stage data are described in the Discussion section. We also revised the manuscript as you pointed out (lines 270-273).<br><br>Why is this important for clinical outcomes? Is there any data regarding how many cervical cancers were missed as a result of delayed screening (either from the authors data set or from any of the references)?<br>The five-year overall survival rates for cervical cancer stages I and II are known to be approximately 90% and 75%, respectively. Early detection by periodic screening is critical in order to prevent death from cervical cancer. If regular screening is skipped as a result of the earthquake, it may be detected later as advanced cancer. This condition was found in the area that was impacted by Hurricane Katrina.We added this in the Discussion section (lines 273-282).<br><br>Lines 235-236 contain repeated information which was just mentioned in lines 232-234.<br>We deleted this sentence in the revised manuscript.<br><br>Lines 239-242 need to be written with a more clear introduction or transition. Lines 241-242 are unclear.<br>It is known that smoking increases the risk of HPV infection. In this sentence, it suggests the number of smokers is increasing in many affected areas, implying that cervical cancer should be considered in these areas. However, there is no direct evidence of smoking rates in coastal and non-coastal areas surveyed in this research. Therefore, we removed this information from the revised manuscript.<br><br>Consider writing a separate conclusion since lines 244-248 do not transition in a clear way since the discussion prior to it pertains to smoking a mental stress.<br>We removed the description of mental stress and smoking and added a separate conclusion in the revised manuscript.<br><br>The conclusive remark (line 246-248) is meaningful however there is lack of data to support this claim. The authors should consider including evidence from the literature supporting why design and construction of a comprehensive medical system etc... would be beneficial.<br>During the sub-acute and chronic phases following the Great East Japan Earthquake, there were significant needs for medical and public health assistance that included infectious disease control and mental health care at evacuation facilities. The provision of health and health facilities is a priority issue in the international guidelines on disaster reduction actions for the 15 years to 2030 adopted by the 3rd United Nations

Powered by Editorial Manager® and ProduXion Manager® from Aries Systems Corporation

	World Conference on Disaster Reduction (WCDRR, 2015). The present study suggests the importance of prompt reconstruction of healthcare systems and the inclusion of cancer screening in these systems to maintain women's health. We have described this in the Discussion section as a conclusion (lines 306-314).<br><br>To Reviewer #3:<br>We appreciate your comments regarding our manuscript entitled “Cervical cancer screening rates before and after the Great East Japan Earthquake in Miyagi prefecture, Japan” (manuscript ID: PONE-D-19-25407). We have revised the manuscript according to your comments and provided detailed responses to specific comments below.<br><br>The study utilized appropriate statistics in analyzing data collected from relevant sources. The conclusions drawn and recommendations made were based on the results of the study. However, the following observations and comments addressed.<br><br>1. The cervical cancer screening rate for Japan should be stated for adequate comparison with other countries (lines 40-41).<br>We have added cervical cancer screening rates for Japan (33.7%; age group: 20-69 years) and Miyagi Prefecture (42.1%; age group: 20-69 years) (lines 46-50).<br><br>2. Recast the sentence on lines 50 - 51 removing 'screening for cervical cancer screening' so the sentence reads 'All mobile van screenings are population-based'.<br>We appreciate your comments. We have revised this sentence accordingly.<br><br>3. Replace 'as per' with a standard English word or phrase (line 65).<br>This phrase was considered meaningless and has been removed from the revised manuscript.<br><br>4. Replace 'i.e' with the appropriate words (line 97).<br>This word has been corrected to "e.g." (line 113).<br><br>5. Lines 196 - 197: Report in the past.<br>We appreciate your comment. This sentence was deleted due to the other reviewers' suggestions.<br><br>References.<br>6. Cross-check if the following articles are single-paged articles: reference nos [3], [14], [21], [39]; and provide the complete pages where missing.<br>These references are single-paged online journals.<br><br>7. Provide the year of publication for no [5], and page numbers for no [27]. Also check the correctness of the page number for reference no [38].<br>Reference No. [5] was 2016 public data of National Cancer Center in Japan. Nos. [27] and [28] are also online journals, each having a single number "4" and "e018943".
**Additional Information:**	
**Question**	**Response**
**Financial Disclosure**<br><br>Enter a financial disclosure statement that describes the sources of funding for the work included in this submission. Review the submission guidelines for detailed requirements. View published research articles from *PLOS ONE* for specific examples.<br><br>This statement is required for submission and **will appear in the published article** if	The authors received no specific funding for this work.

the submission is accepted. Please make sure it is accurate.<br><br>**Unfunded studies**<br>Enter: *The author(s) received no specific funding for this work.*<br><br>**Funded studies**<br>Enter a statement with the following details:<br>• Initials of the authors who received each award<br>• Grant numbers awarded to each author<br>• The full name of each funder<br>• URL of each funder website<br>• Did the sponsors or funders play any role in the study design, data collection and analysis, decision to publish, or preparation of the manuscript?<br>• **NO** - Include this sentence at the end of your statement: *The funders had no role in study design, data collection and analysis, decision to publish, or preparation of the manuscript.*<br>• **YES** - Specify the role(s) played.<br><br>* typeset	
**Competing Interests**<br><br>Use the instructions below to enter a competing interest statement for this submission. On behalf of all authors, disclose any competing interests that could be perceived to bias this work—acknowledging all financial support and any other relevant financial or non-financial competing interests.<br><br>This statement **will appear in the published article** if the submission is accepted. Please make sure it is accurate. View published research articles from *PLOS ONE* for specific examples.	The authors have declared that no competing interests exist.

**NO authors have competing interests**<br><br>Enter: *The authors have declared that no competing interests exist.*<br><br>**Authors with competing interests**<br><br>Enter competing interest details beginning with this statement:<br><br>*I have read the journal's policy and the authors of this manuscript have the following competing interests: [insert competing interests here]*<br><br>* typeset	
**Ethics Statement**<br><br>Enter an ethics statement for this submission. This statement is required if the study involved:<br><br>• Human participants<br>• Human specimens or tissue<br>• Vertebrate animals or cephalopods<br>• Vertebrate embryos or tissues<br>• Field research<br><br>Write "N/A" if the submission does not require an ethics statement.<br><br>General guidance is provided below. Consult the submission guidelines for detailed instructions. **Make sure that all information entered here is included in the Methods section of the manuscript.**	This research was approved by the ethics committees of the International Research Institute of Disaster Science, Tohoku University (No.2019-006) and Miyagi Cancer Society (No.1904).

Powered by Editorial Manager® and ProduXion Manager® from Aries Systems Corporation

**Format for specific study types**<br><br>**Human Subject Research (involving human participants and/or tissue)**<br>• Give the name of the institutional review board or ethics committee that approved the study<br>• Include the approval number and/or a statement indicating approval of this research<br>• Indicate the form of consent obtained (written/oral) or the reason that consent was not obtained (e.g. the data were analyzed anonymously)<br><br>**Animal Research (involving vertebrate animals, embryos or tissues)**<br>• Provide the name of the Institutional Animal Care and Use Committee (IACUC) or other relevant ethics board that reviewed the study protocol, and indicate whether they approved this research or granted a formal waiver of ethical approval<br>• Include an approval number if one was obtained<br>• If the study involved *non-human primates*, add *additional details* about animal welfare and steps taken to ameliorate suffering<br>• If anesthesia, euthanasia, or any kind of animal sacrifice is part of the study, include briefly which substances and/or methods were applied<br><br>**Field Research**<br><br>Include the following details if this study involves the collection of plant, animal, or other materials from a natural setting:<br>• Field permit number<br>• Name of the institution or relevant body that granted permission	
**Data Availability**<br><br>Authors are required to make all data underlying the findings described fully available, without restriction, and from the time of publication. PLOS allows rare exceptions to address legal and ethical concerns. See the PLOS Data Policy and FAQ for detailed information.	Yes - all data are fully available without restriction

Powered by Editorial Manager® and ProduXion Manager® from Aries Systems Corporation

A Data Availability Statement describing where the data can be found is required at submission. Your answers to this question constitute the Data Availability Statement and **will be published in the article**, if accepted.<br><br>**Important:** Stating 'data available on request from the author' is not sufficient. If your data are only available upon request, select 'No' for the first question and explain your exceptional situation in the text box.<br><br>Do the authors confirm that all data underlying the findings described in their manuscript are fully available without restriction?	
**Describe where the data may be found in full sentences. If you are copying our sample text, replace any instances of XXX with the appropriate details.**<br><br>• If the data are **held or will be held in a public repository**, include URLs, accession numbers or DOIs. If this information will only be available after acceptance, indicate this by ticking the box below. For example: *All XXX files are available from the XXX database (accession number(s) XXX, XXX.*).<br>• If the data are all contained **within the manuscript and/or Supporting Information files**, enter the following: *All relevant data are within the manuscript and its Supporting Information files.*<br>• If neither of these applies but you are able to provide **details of access elsewhere**, with or without limitations, please do so. For example:<br><br>*Data cannot be shared publicly because of [XXX]. Data are available from the XXX Institutional Data Access / Ethics Committee (contact via XXX) for researchers who meet the criteria for access to confidential data.*<br><br>*The data underlying the results presented in the study are available from (include the name of the third party*	All relevant data are within the manuscript and its Supporting Information files.

and contact information or URL).<br>• This text is appropriate if the data are owned by a third party and authors do not have permission to share the data.<br><br>* typeset	
Additional data availability information:	

Powered by Editorial Manager® and ProduXion Manager® from Aries Systems Corporation

Manuscript
Click here to access/download;Manuscript;cancer_screening__Revised_Fin

Cervical cancer screening rates before and after the Great East Japan Earthquake in the Miyagi Prefecture, Japan

Yasuhiro Miki[1], Toru Tase[2], Hideki Tokunaga[3], Nobuo Yaegashi[3], Kiyoshi Ito[1,4*]

[1] Department of Disaster Obstetrics and Gynecology, International Research Institute of Disaster Science, Tohoku University, Sendai, Japan.

[2] Cancer Detection Center, Miyagi Cancer Society, Sendai, Japan.

[3] Department of Obstetrics and Gynecology, Tohoku University Graduate School of Medicine, Sendai, Japan.

[4] Disaster Medical Science Group, Core Research Cluster of Disaster Science, Tohoku University, Sendai, Japan.

Running Title: Cervical cancer screening and the Great East Japan Earthquake

*Corresponding author:

E-mail: kito@med.tohoku.ac.jp (KI)

Abstract

After disasters, issues pertaining to women’s health such as irregular periods and bleeding are well surveyed. However, the management of women’s health, especially changes in the rate of health checkups, has not been investigated. In the present study, we focused on the change in the cervical cancer screening rates (CCS-Rs) before and after the Great East Japan Earthquake in Miyagi Prefecture, Japan. The earthquake had a magnitude of 9.0, a profound disaster. We examined the CCS-R from 2009 to 2016 in 45 areas of the Miyagi Prefecture. Screening was completed using mobile vans. In the 4 areas impacted by the tsunami after the earthquake, a marked decrease in the CCS-R was observed in 2011 when the earthquake took place (more than a 3% decrease compared with that in the previous year). The CCS-Rs in these 4 regions remained lower in 2016 than in the previous year. In 2009–2016 except for 2014, CCS-Rs in coastal areas (9 areas) were significantly lower than those in the non-coastal areas (36 areas). A delay in seeking healthcare, also known as “patient’s delay,” is considered as one of the problems of cancer treatment in affected areas. It is possible that a decrease in the CCS-R may lead to low detection of advanced stages of cancer. Therefore, the establishment of a comprehensive medical system including medical screening after a disaster is important for the management of women’s health.

Introduction

The cervical cancer incidence in Japan is 14.7 (age-standardized rates per 100,000 population), which is higher than that in other developed countries such as United States (6.5), Australia (6.0), and South Korea (8.4) and similar to that in India (14.7) and the Philippines (14.9) (Data from Cancer Today - IARC, https://gco.iarc.fr/today/online-analysis-table, accessed 2020.1.17). Furthermore, vaccination coverage in Japan (under 1%) is significantly lower than that in other countries where cervical cancer vaccines are licensed [1,2]. Therefore, cervical cancer screening is considered crucial. However, the screening rate in Japan is considerably lower than that in Western and other countries [3]. It is reported that the screening rates in the United States and Britain and those in France and South Korea are approximately 80% and 70%, respectively [3]. Furthermore, the screening rate in Japan (33.7% [age group: 20-69 years]) is lower than that in Australia (56%) [3,4,5]. An increase in the coverage of women receiving periodic cervical cancer screening is urgently needed in Japan. The rate of cervical cancer screening in the Miyagi Prefecture (42.1%; age group 20-69 years) was the second highest among the 47 prefectures in Japan in 2016 [5]. In the Miyagi Prefecture, the mobile van service (or mobile examination unit) offering cervical cancer screening serves rural areas where it is difficult to obtain an examination

in a hospital or medical center [6,7]. There are two types of cancer screenings: population-based screening and opportunistic screening such as private and company medical check-ups. All of the mobile van screenings are population-based screenings.

On March 11, 2011, a magnitude 9.0 earthquake, termed the Great East Japan Earthquake, struck the east area of Japan. The abbreviation “3.11” has been used for this earthquake, mainly by the Japanese mass media as the earthquake occurred on March 11, 2011. After the Great East Japan Earthquake, higher-than-expected tsunami waves hit coastal zones of Tohoku and caused devastating damage. The economic loss was serious, and the damage caused by the earthquake was estimated to be approximately 16–25 trillion yen [8]. The tsunami-impacted area was predicted to require 10 years for reconstruction [9]. In the Miyagi Prefecture, a mobile van service offering cervical cancer screening covered the areas severely affected by the tsunami after the Great East Japan Earthquake. The screening resumed promptly to maintain the health of the residents in the disaster area. In many areas, cervical cancer screening was resumed in April of the year of the disaster, 2011. However, in coastal areas (L-2, L-3, T-3, U-1b in Fig. 1), the re-initiation of screenings was delayed from July to December, 2011. The screening was resumed only in Oshika (L-7 in Fig. 1) in February, 2012 following the earthquake. Unfortunately, the people affected by the disaster continue to be mentally

and financially unstable and cannot afford to maintain their health [10,11,12].

It is well known that conflicts and disasters have a major impact on healthcare and result in delays in the diagnosis and treatment of patients with cancer [10,11]. Furthermore, the social isolation caused by a disaster may delay the opportunity to examine and treat patients with cancer [12]. A study at a city hospital in 2005 in New Orleans, affected by Hurricane Katrina, found that the mean time from last cytology (Pap test) to diagnosis of cervical cancer was significantly longer after the hurricane (7.7 years) than before the hurricane (4.2 years) (Abstract of the 2001 Western Association of Gynecologic Oncologists Annual Meeting, doi: 10.1016/j.ygyno.2011.07.082 [13]). In addition, the clinical stage of cervical cancer at diagnosis was significantly higher after the hurricane than before the hurricane [13]. Women's health hazards such as abnormal menstrual cycles, pelvic inflammation, and lower genital tract infections after disasters also have been reported in various countries [14-18]. Although the failure of health management is considered to lead to long-term health risks, the cervical cancer screening rate (CCS-R) after a disaster remains unexamined. In this study, we examined the trend of CCS-Rs from the Japan fiscal year (April 1 to March 31) 2009–2016 in 45 areas of the Miyagi Prefecture where cervical screenings were performed by the Miyagi Cancer Society (Fig 1, S1 Table).

Fig 1. The Tohoku region in Japan and the survey area in the Miyagi Prefecture. (A) The Tohoku Region has six prefectures: Aomori Prefecture, Iwate Prefecture, Miyagi Prefecture, Akita Prefecture, Yamagata Prefecture, and Fukushima Prefecture. (B) The survey area in Miyagi. Enlarged views are presented for each of the areas a, b, and c. Also, see S1 Table for details. Bold style code numbers represent areas where cervical cancer screenings were performed by mobile vans.

Materials and methods

CCS-Rs were obtained from the Annual Report in 2009–2016 and were compiled by the Miyagi Cancer Society (Sendai, Miyagi, Japan). The Miyagi Cancer Society was established as a public interest incorporated foundation and has formed a cancer screening and cancer registration business in Miyagi Prefecture, Japan. The annual report is also compiled as one of the functions of the Miyagi Cancer Society. The population of each region in each year was also extracted from this Annual Report.

The incidence of cervical cancer has increased in Japanese women in their mid-20s [19]. Therefore, Japanese cervical cancer screening guidelines recommend to

begin screening at the age of 20 years [20]. The CCS-R was defined by the following equation: CCS-R = {(Number of women aged 20 years and older who underwent cervical cancer screening) ÷ (Total population of women aged 20 years and older)} × 100 (%). The difference in CCS-R for each year compared with the year before the Great East Japan Earthquake was calculated as follows: Δ[CCS-R in the year] = {CCS-R in the year (%)} − {CCS-R in the year before the Great East Japan Earthquake, 2010 (%)}; *e.g.*, Δ[CCS-R 2011] (%) = CCS-R in 2011 (%) − CCS-R in 2010 (%). The difference in population in each area was calculated similarly: Δ [Pop. in the year] = 100 (%) − {(Pop. in the year) ÷ (Pop. in the year before the earthquake, 2010) × 100 (%)}.

In the Miyagi Prefecture, cervical cancer screening was performed in the mobile van or the medical center/hospital. The medical exam statistics at the medical center or hospitals in the Miyagi Prefecture included a large number of sporadic outpatients, who complained of symptoms such as irregular bleeding. Therefore, the results of screening at medical facilities (23 areas) are shown in S1 Figure. The study area (45 areas) is shown in Figure 1 and S1 Table. In those regions, cervical cancer screening was provided by a mobile van. All of the data were processed using Microsoft Excel (Microsoft Corporation, Redmond, WA, USA). Mann–Whitney U test with StatView ver. 5.0 (SAS Institute Inc., San Francisco, CA, USA) was used for comparison between the

groups. This research was approved by the ethics committees of the International Research Institute of Disaster Science, Tohoku University (Sendai, Japan) and Miyagi Cancer Society (Sendai, Japan).

Results

Differences in the CCS-R in each region from 2010 to 2016 are summarized in Table 1. Among the areas covered by the mobile van, the Δ[CCS-R 2011] in the 4 regions markedly decreased (over −3.0%) (Fig 2A): Ogatsu −5.2% (L-3), Onagawa −7.0% (N-1), Karakuwa −4.8% (T-2), and Shizugawa −4.1% (U-1a). These four coastal regions were affected by the tsunami. In Ogatsu (L-3), the CCS-Rs tended to recover the year after the earthquake (2013) and decreased approximately by 2.5% in 2013–2015 compared with the rate one year prior to the earthquake (2010). However, the Δ[CCS-R 2016] was −4.1% (Fig 2Ab). The population from 2011 (Δ[Pop.2011]) to 2016 (Δ[Pop.2016]) in Ogatsu (L-3) remained at −30.3% to −56.8% (Fig 2Aa). In Onagawa (N-1), where the decrease in the CCS-R was the highest in the year the earthquake occurred (Δ[CCS-R 2011], −7.0%), a high reduction ratio (−5.2 → −5.0 → −5.6 → −6.9 → −4.5) was observed even after the earthquake from (Δ[CCS-R 2012] to

Δ[CCS-R 2016]) (Fig 2Ab). The Δ[Pop.2011] to Δ[Pop.2016] in Onagawa (N-1) remained at −20.6% to −34.8% (Fig 2Aa). In Karakuwa (T-2), although the CCS-R recovered to a decreased rate of less than 2.0% (Δ[CCS-R 2012]), it decreased by 3.0% from (Δ[CCS-R 2013] to Δ[CCS-R 2016]) (Fig 2Ab). The Δ[Pop.2011] to Δ[Pop.2016] in Karakuwa (T-2) remained at −4.0% to −11.1% (Fig 2Aa). In Shizugawa (U-1a), the CCS-R decreased by 3% in the year following the earthquake (Δ[CCS-R 2012]) but recovered to the level one year before the earthquake (2010) in 2013 after the merger with Utatsu (U-1b) and increased by more than 3.0% in 2015 and 2016 (Table 1). The Δ[Pop.2011] to Δ[Pop.2016] in Minamisanriku (U-1 = U-1a + U-1b) remained at −10.5% to −19.1% (Table 1).

Table 1. Differences in the Cervical Cancer Screening Rates Between Before and After the Great East Japan Earthquake**.**

**Code No.**			**2010–2009**	**2011–2010**	**2012–2010**	**2013–2010**	**2014–2010**	**2015–2010**	**2016–2010**
B	1	Zao Town	1.5	−0.8	−1.8	−1.0	−1.8	−0.6	−1.7
	2	Shichigasyuku Town	−1.4	0.0	−1.7	−1.7	***−4.5***	***−3.3***	***−3.4***

D	1	Marumori Town	0.7	−0.2	0.9	−0.1	0.1	1.8	0.5
E	3	Murata Town	0.4	0.5	−0.4	0.5	0.7	0.8	1.4
	4	Kawasaki Town	***6.4***	***3.8***	***4.5***	***3.2***	***4.7***	***7.3***	***6.2***
H	2	Yamamoto	1.0	−0.8	−1.1	−1.7	−0.7	−0.9	−0.9
L	2	Kahoku	0.3	−1.1	−1.8	−1.9	−0.9	−1.5	***−3.9***
	3	Ogatsu	0.2	***−5.2***	***−4.0***	−2.4	−2.6	−2.4	***−4.1***
	5	Monou	0.3	−0.6	−2.3	***−3.4***	−2.7	***−3.5***	***−5.6***
	6	Kitakami	0.3	1.3	0.0	−1.0	0.6	−2.2	***−4.3***
	7	Oshika	−0.8	−2.9	−1.9	−1.7	0.7	***−3.6***	−1.8
N	1	Onagawa Town	0.8	***−7.0***	***−5.2***	***−5.0***	***−5.6***	***−6.9***	***−4.5***
O	3	Sambongi	−0.6	1.7	1.7	1.7	***−4.3***	1.4	2.2
	4	Kashimadai*	1.4	0.5	1.4	1.1	1.3	0.8	2.0
	5	Iwadeyama†	0.6	1.3	1.6	1.3	1.8	2.0	2.8
	6	Naruko	0.2	1.1	0.8	−0.9	0.2	−1.4	−0.1
	7	Taziri	2.1	0.9	0.5	0.6	1.2	0.3	2.0
P	1	Misato Town	0.2	0.1	−1.0	0.0	−0.8	2.6	2.1

		Kogota							
	2	Misato Town Nango	0.5	−1.9	−2.4	−1.8	−2.2	0.5	−1.3
	3	Wakuya Town‡	***3.9***	−1.2	−2.9	−1.5	−1.2	−1.9	−0.9
Q	1	Kami Town	−0.7	0.5	0.4	2.1	0.9	1.8	***3.9***
	2	Shikama Town†	−1.4	1.4	2.3	***3.2***	***4.7***	***4.7***	***4.7***
R	2	Ichihasama	0.8	−0.9	−0.4	−0.6	−0.4	−2.9	0.6
	3	Takashimizu	1.4	0.3	−0.8	1.5	1.4	1.4	2.0
	4	Semine	1.3	−1.0	−1.3	−2.5	−0.3	1.1	0.5
	5	Shiwahime	1.3	−0.5	−1.3	−2.1	−0.8	0.4	−0.4
	6	Hanayama	0.9	−1.7	−1.2	0.0	−2.3	0.4	−1.2
	8	Kurikoma	1.0	0.1	−1.1	−0.8	−0.6	−0.2	−0.6
	9	Uguisusawa	1.0	0.1	−0.2	−1.0	−1.1	−0.8	−0.5
	10	Kannari	0.3	0.3	−0.3	−0.4	−0.2	−1.4	−0.1
S	1	Toyoma	0.4	−0.4	0.2	−0.2	−0.5	−0.1	0.1
	3	Towa	−0.3	0.0	1.8	0.8	1.6	2.7	2.3

	4	Nakada	1.3	−0.7	2.4	0.2	0.1	0.8	−0.1
	5	Toyosato	−0.7	1.0	2.3	***3.0***	2.6	***4.2***	***3.3***
	6	Yoneyama	−0.5	1.5	2.0	2.3	1.3	1.1	0.8
	7	Minamikata	0.1	−0.8	−0.3	−0.2	−1.4	−1.4	−2.0
	8	Ishikoshi	1.4	−1.6	−0.2	0.0	0.2	−1.2	−0.1
	9	Tsuyama	1.5	−1.9	−0.8	−0.5	0.3	1.6	2.3
T	2	Karakuwa	−0.3	***−4.8***	−1.9	−2.7	−2.5	***−3.5***	−2.7
	3	Motoyoshi	1.9	−2.3	−1.3	−1.1	−1.8	−2.6	−2.6
U	1 a	Shizugawa	0.3	***−4.1***	***−3.1***	1.7	2.7	***3.6***	***3.1***
	1 b	Utatsu	−0.4	−2.7	***−4.1***	–	–	–	–
V	2	Ohsato Town	−0.2	−0.4	−0.7	1.0	1.1	0.0	0.2
	3	Ohira Village	0.2	−0.6	0.0	−0.4	−0.4	−0.4	1.0
W	1	Tomiya City	1.4	−0.3	−2.6	−1.2	−2.0	***−3.6***	−1.9

Areas covered by mobile vans were examined. Screening by the mobile vans *since 2015, †since 2013, and ‡since 2011; Bold italic, change of 3% or more; U-1, Shizugawa (U-1a) and Utatsu (U-1b) were integrated in 2013. The 2010 cervical cancer screening rate was compared with that of 2009.

Fig 2. Change in the CCS-R in coastal areas.

(A) a. Coastal areas showing a remarkable decrease in the cervical cancer screening rates (CCS-Rs) in the year following the Great East Japan Earthquake (over −3.0%). The bar graph shows the CCS-R (%), and the line graph shows the population (number of people). b. Differences in the CCS-R between before and after the Great East Japan Earthquake in each area demonstrated in “a”. *, change of 3% or more. U-1 since Shizugawa (U-1a) and Utatsu (U-1b) were integrated in 2013; the data for 2009–2012 include a total of two regions.

(B) a. Change in CCS-R in other coastal areas. The bar graph shows the CCS-R (%), and the line graph shows the population (number of people). b. Differences in the CCS-R between before and after the Great East Japan Earthquake in each area demonstrated in “a”. *, change of 3% or more.

In the tsunami-affected coastal areas other than the above four towns, the Δ[CCS-R 2011] was as follows (Fig 2B): Yamamoto−0.8% (H-2), Kahoku −1.1% (L-2), Kitakami +1.3% (L-6), Oshika −2.9% (L-7), and Motoyoshi −2.3% (T-3) (Fig 2Bab). The Δ [Pop. 2011] was as follows; Yamamoto −11.3% to −22.4% (H-2), Kahoku −3.5%

to −7.0 (L-2), Kitakami −15.3% to −31.7% (L-6), Oshika −14.0% to −38.7% (L-7), and Motoyoshi −3.7% to −6.4% (T-3) (Fig 2Ba).

In non-coastal areas, the CCS-R declined by more than 3.0% compared with that of the year before the earthquake during the survey period in four places [Shichigashuku (B-2), Kahoku (L-2), Monou (L5), Tomiya (W-1)] (Fig 3A). In contrast, in the 4 non-coastal regions, the CCS-R increased by more than 3.0% during the survey period compared with that before the earthquake year [Kawasaki (E-4), Kami (Q-1), Shikama (Q-2), Tomiya (S-5)] (Fig 3B). No notable increase or decrease (±3.0%) was observed in other non-coastal areas (Fig 4).

Fig 3. Change in the cervical cancer screening rate (CCS-R) in non-coastal areas examined in this study.

(A) a. The CCS-R declined by more than 3.0% compared with that of the year before the earthquake (over −3.0%). The bar graph shows the CCS-R (%), and the line graph shows the population (number of people). b. Differences in the CCS-R between before and after the Great East Japan Earthquake in each area demonstrated in “a”. *, change of 3% or more.

(B) a. The CCS-R declined by more than 3.0% compared with that of the year before

the earthquake (over +3.0%). The bar graph shows the CCS-R (%), and the line graph shows the population (number of people). b. Differences in the CCS-R between before and after the Great East Japan Earthquake in each area demonstrated in “a”. *, change of 3% or more.

Fig 4. Change in the cervical cancer screening rate (CCS-R) in other non-coastal areas examined in this study.

The bar graph shows the CCS-R (%), and the line graph shows the population (number of people).

When the areas examined were divided into coastal and non-coastal areas, the CSS-R was significantly lower in the coastal areas during 2011 ($p = 0.0046$), 2012 ($p = 0.0020$), 2013 ($p = 0.0247$), 2015 ($p = 0.0370$), and 2016 ($p = 0.0135$) (Fig 5). No significant difference was found in the 2-year comparison before the earthquake (2010–2009, $p = 0.2018$) and 2014 (2014–2010, $p = 0.6303$) (Fig 5).

Fig 5. Comparison of the change in the cervical cancer screening rate in coastal

and non-coastal regions.

The bar graph shows the median value of differences in the CCS-R between before and after the Great East Japan Earthquake (%). Coast, nine coastal regions; Non-coasts, 36 non-coastal regions.

Discussion

The health of women, especially pregnant women, has been investigated in the areas affected by the Great East Japan Earthquake [21-23]; however, no research has been conducted with regard to cancer screening. In the present study, we first clarified the difference in the CCS-R of the Miyagi Prefecture before and after the Great East Japan Earthquake. In the present study, after the Great East Japan Earthquake, the CCS-R significantly decreased in the coastal areas compared with that in other areas. The coastal areas in the Miyagi Prefecture were severely impacted by an unforeseen tsunami. Even within the screening areas, a drastic population decline was observed immediately after the earthquake. It is well known that the tsunami resulted in severe human and residential damage as a result of the Great East Japan Earthquake [24,25]. Therefore, coastal populations are considered to have decreased both due to death as

well as migration as a consequence of the tsunami. After the Great East Japan Earthquake, the population also decreased in areas with a severe decrease in the CCS-R. Although the decrease in the CCS-R could have been attributed to the decline in the population, there were many areas in which there was no clear relationship between the decrease in population and the CCS-R. In the present study, we used the total population of each region for evaluation. We found that further studies that are corrected for the population of women aged over 20 years are required. The economic loss caused by the Great East Japan Earthquake in the Miyagi Prefecture was more severe in coastal areas than in inland areas (4.9 vs 1.6 trillion yen) in 2012 [24]. In coastal areas, the damage to livelihoods and social infrastructure resulted in a loss of 2.0 trillion yen and the loss due to residential damage was 1.4 trillion yen. This was 2.4- and 36.2-times higher than that in inland areas, respectively [24]. Furthermore, reconstruction in the Miyagi Prefecture was reported to be markedly lower in coastal areas than in inland areas in 2012 to 2016 [25].

From the year after the Great East Japan Earthquake to 2016, there were some areas where the CCS-R recovered; however, other areas did not show such recovery. Furthermore, in many areas where cervical cancer screening had been conducted at medical facilities by the Miyagi Cancer Society, a decline in the CCS-R was observed in

the year following the earthquake (S1 Figure). In an evidence review by CPSTF (USA), small media sources such as newspapers and educational videos as well as solicitation by telephone or letter are recommended as interventions to increase the CCS-R (https://www.thecommunityguide.org/content/task-force-findings-cancer-prevention-and-control; accessed 2020.1.17). In Japan, it was also reported that local government initiatives and education contributed to increasing the screening rate of young women [26]. However, a detailed investigation is required in areas where these screening rates have declined and awareness attempts by local government and other organizations may have been delayed. It is important for future post-disaster measures to clarify the details of the recruitment of cervical cancer screening programs for women in each area.

It is well known that conflicts and disasters have a major influence on healthcare and cause delays in diagnosis and treatment of patients with cancer [10]. In the Fukushima Prefecture, which was impacted by a triple disaster (an earthquake, a tsunami, and a nuclear accident), delays in symptom recognition to the first medical examination in patients with breast cancer have been reported [11]. The trend of risk due to patients' delay continues even following the disaster [11]. It is also reported that social isolation due to the triple disaster in Fukushima may have delayed opportunities for medical consultation and appropriate therapy among patients with colon cancer [12].

A survey of 366 patients with cervical cancer from urban hospitals in New Orleans, USA affected by Hurricane Katrina in 2005 was conducted [13]. The mean time from last cytology (Pap test) to diagnosis of cervical cancer was significantly longer after the hurricane (7.7 years) than before the hurricane (4.2 years) [13]. In addition, the cancer stage was significantly higher ($p < 0.01$) after the hurricane (From 2006 to 2010: stage I, 40 cases; stage II, 36 cases; stage III, 32 cases; stage IV, 32 cases.) than before the hurricane (From 2000 to 2005: stage I, 101 cases; stage II, 60 cases; stage III, 37 cases; stage IV, 28 cases) [13]. The five-year overall survival rates for cervical cancer stages I and II are known to be approximately 90% and 75%, respectively [19]. Early detection by periodic screening is critical in order to prevent death from cervical cancer [20]. If regular screening is skipped as a result of the earthquake, it may be detected later as advanced cancer. This situation was found in the area that was impacted by Hurricane Katrina [13]. These reports illustrate the importance of prompt reconstruction of healthcare systems, including screening, for early detection [13,27]. Compared to the USA CCS-R, the Japan CCS-R is too low [3,5]. Therefore, it would be difficult to perform the same analysis as that in US research due to the small number of comparative patients. Otherwise, in Japan, cervical cancer screening is expected to reduce cervical cancer deaths (recommendation grade B), with evidence levels of 2++

for conventional and 2+ for liquid-based cytology [20]. There are limitations to conducting a further detailed analysis in the areas examined in this study; however, a reduction in CCS-Rs suggests that women's health will be significantly affected.

The trauma experienced during tsunamis remains a strong psychological stressor among disaster victims [28-32]. A survey after the Great East Japan Earthquake reported that difficulty settling back into daily living, the presence of preexisting illnesses, and the disruption of social networks were the most important risk factors for mental health disorders. These conditions did not improve even 2 years after the disaster [33]. Therefore, it is possible that women in the affected areas will continue to be exposed to long-term distress. Psychological stress is believed to exhibit immunosuppressive effects through the inhibition of several lymphocyte functions. Stress is known to be involved in tumorigenesis or tumor development by the inactivation of cytotoxic T cells and natural killer (NK) cells [34]. In surveys after natural disasters such as earthquakes and hurricanes, associations between disaster-related stress and decrease of immune functions, especially suppression of NK cell activity, have been reported [35-37]. A survey of victims of the 1995 Southern Hyogo Prefecture Earthquake in Japan revealed that post-traumatic stress disorder symptoms and poor lifestyle are associated with low NK cell activity [37].

Reconstruction of the healthcare system after the Great East Japan Earthquake is significant; however, the progress is uneven among the affected areas [38]. Design and construction of a comprehensive medical system, including lifestyle habit improvements, mental health care, and medical screening after a disaster, are essential for preventing health damage to women. During the sub-acute and chronic phases following the Great East Japan Earthquake, there were significant needs for medical and public health assistance that included infectious disease control and mental health care at evacuation facilities [39]. The provision of health and health facilities is a priority issue in the international guidelines on disaster reduction actions for the 15 years to 2030 adopted by the 3rd United Nations World Conference on Disaster Reduction (WCDRR, 2015) [40]. The present study suggests the importance of prompt reconstruction of healthcare systems and the inclusion of cancer screening in these systems to maintain women's health.

References

[1] Brotherton JML, Bloem PN. Population-based HPV vaccination programmes are safe and effective: 2017 update and the impetus for achieving better global coverage. Best Pract Res Clin Obstet Gynaecol. 2018;47:42-58.

[2] Ikeda S, Ueda Y, Yagi A, Matsuzaki S, Kobayashi E, Kimura T, et al. HPV vaccination in Japan: what is happening in Japan? Expert Rev Vaccines. 2019;18(4):323-325.

[3] Komiyama M, Hasegawa K. Comparison of preventive care for cervical cancer between Japan and western countries: A review. J Pharma Care Health Sys. 2017;4: 185.

[4] Australian Institute of Health and Welfare 2018. Cervical screening in Australia 2018. Cat. no. CAN 111. Canberra: AIHW.

[5] Cancer Registry and Statistics. Cancer Information Service, National Cancer Center, Japan.

[6] Yajima A, Higashiiwai H, Sato A, Watanabe M, Hoshi K, Mori T, et al. Mass population screening for cervical cancer in Miyagi Prefecture. Gynecol Oncol. 1979;8: 131-140.

[7] Sato S, Matsunaga G, Konno R, Yajima A. Mass screening for cancer of the uterine cervix in Miyagi Prefecture, Japan. Effects and problems. Acta Cytol. 1998;42: 299-304.

[8] Ikeda, Maria M., and Miharu Matsumaru. Leadership and social innovation

initiatives at the grassroots during crises. Japan Social Innovation Journal 2.1 2012: 77-81.

[9] Koshimura S, Shuto N. Response to the 2011 Great East Japan Earthquake and Tsunami disaster. Philos Trans A Math Phys Eng Sci. 2015;373 https://doi.org/10.1098/rsta.2014.0373.

[10] El Saghir NS, Soto Pérez de Celis E, Fares JE, Sullivan R. Cancer care for refugees and displaced populations: Middle East conflicts and global natural disasters. Am Soc Clin Oncol Educ Book. 2018; 433-440.

[11] Ozaki A, Nomura S, Leppold C, Tsubokura M, Tanimoto T, Yokota T, et al. Breast cancer patient delay in Fukushima, Japan following the 2011 triple disaster: a long-term retrospective study. BMC Cancer. 2017;17: 423.

[12] Ozaki A, Leppold C, Sawano T, Tsubokura M, Tsukada M, Tanimoto T, et al. Social isolation and cancer management - advanced rectal cancer with patient delay following the 2011 triple disaster in Fukushima, Japan: a case report. J Med Case Rep. 2017;11: 138.

[13] Kanjanvaikoon P. Gerber D. Robison WR. Long term impact of natural disaster on cervical cancer demographics. Gynecol Oncol. 2011;123: 446 (Meeting report).

[14] Liu S, Han J, Xiao D, Ma C, Chen B. A report on the reproductive health of women after the massive 2008 Wenchuan earthquake. Int J Gynaecol Obstet. 2010;108: 161-164.

[15] Li XH, Qin L, Hu H, Luo S, Li L, Fan W, et al. Influence of the Wenchuan earthquake on self-reported irregular menstrual cycles in surviving women. Gynecol Endocrinol. 2011;27: 706-710.

[16] Bloem CM, Miller AC. Disasters and women’s health: reflections from the 2010 earthquake in Haiti. Prehosp Disaster Med. 2013;28: 150-154.

[17] Sohrabizadeh S, Tourani PhD S, Khankeh HR. Women and health consequences of natural disasters: Challenge or opportunity? Women Health. 2016;56: 977-993.

[18] Goodman A. In the aftermath of disasters: the impact on women’s health. Crit Care Obst Gyne. 2016;2: 6.

[19] Yamagami W, Nagase S, Takahashi F, Ino K, Hachisuga T, Aoki D, Katabuchi H. Clinical statistics of gynecologic cancers in Japan. J Gynecol Oncol. 2017 Mar;28(2):e32.

[20] Hamashima C, Aoki D, Miyagi E, Saito E, Nakayama T, Sagawa M, et al. Japanese Research Group for Development of Cervical Cancer Screening Guidelines. The

Japanese guideline for cervical cancer screening. Jpn J Clin Oncol. 2010;40: 485-502.

[21] Sato K, Oikawa M, Hiwatashi M, Sato M, Oyamada N. Factors relating to the mental health of women who were pregnant at the time of the Great East Japan earthquake: analysis from month 10 to month 48 after the earthquake. Biopsychosoc Med. 2016;10: 22.

[22] Sugawara J, Hoshiai T, Sato K, Tokunaga H, Nishigori H, Arai T, et al. Impact of the Great East Japan Earthquake on regional obstetrical care in Miyagi Prefecture. Prehosp Disaster Med. 2016;31: 255-258.

[23] Nishigori H, Sugawara J, Obara T, Nishigori T, Sato K, Sugiyama T, et al. Surveys of postpartum depression in Miyagi, Japan, after the Great East Japan Earthquake. Arch Womens Ment Health. 2014;17: 579-581.

[24] Higuchi Y, Inui T, Sugiyama S, Wakabayashi K, Kuga N, Hosoi T, et al. Aftermath of the 3.11 Disaster: An analytical evaluation using official statistics. ESRI Discussion Paper Series No.286; Economic and Social Research Institute Cabinet Office, Tokyo, Japan, 2012.

[25] H30 Survey on Prefectural Attitude by the Miyagi prefecture; 2018, March, in

Japanese.

[26] Ueda Y, Sobue T, Morimoto A, Egawa-Takata T, Hashizume C, Kishida H, Okamoto S, Yoshino K, Fujita M, Enomoto T, Tomine Y, Fukuyoshi J, Kimura T. Evaluation of a free-coupon program for cervical cancer screening among the young: a nationally funded program conducted by a local government in Japan. J Epidemiol. 2015;25(1):50-6.

[27] Man RX, Lack DA, Wyatt CE, Murray V. The effect of natural disasters on cancer care: a systematic review. Lancet Oncol. 2018;19: e482-e499.

[28] Yokoyama Y, Otsuka K, Kawakami N, Kobayashi S, Ogawa A, Tannno K, et al. Mental health and related factors after the Great East Japan earthquake and tsunami. PLoS One. 2014;9: e102497.

[29] Arnberg FK, Bergh Johannesson K, Michel PO. Prevalence and duration of PTSD in survivors 6 years after a natural disaster. J Anxiety Disord. 2013;27: 347-352.

[30] Leiva-Bianchi MC, Araneda AC. Validation of the Davidson Trauma Scale in its original and a new shorter version in people exposed to the F-27 earthquake in Chile. Eur J Psychotraumatol. 2013;4.

[31] Becker SM. Psychosocial care for women survivors of the tsunami disaster in India.

Am J Public Health. 2009;99: 654-658.

[32] Fernando GA. Assessing mental health and psychosocial status in communities exposed to traumatic events: Sri Lanka as an example. Am J Orthopsychiatry. 2008;78: 229-239.

[33] Orui M, Harada S, Hayashi M. Disaster mental health team of the Sendai City Mental Health and Welfare Center. Practical report on long-term disaster mental health services following the Great East Japan Earthquake: Psychological and social background of evacuees in Sendai City in the mid- to long-term post-disaster period. Disaster Med Public Health Prep. 2017;11: 439-450.

[34] Reiche EM, Nunes SO, Morimoto HK. Stress, depression, the immune system, and cancer. Lancet Oncol. 2004;5: 617-625.

[35] Ironson G, Wynings C, Schneiderman N, Baum A, Rodriguez M, Greenwood D, et al. Posttraumatic stress symptoms, intrusive thoughts, loss, and immune function after Hurricane Andrew. Psychosom Med. 1997;59: 128-141.

[36] Segerstrom SC, Solomon GF, Kemeny ME, Fahey JL. Relationship of worry to immune sequelae of the Northridge earthquake. J Behav Med. 1998;21: 433-450.

[37] Inoue-Sakurai C, Maruyama S, Morimoto K. Posttraumatic stress and lifestyles are

associated with natural killer cell activity in victims of the Hanshin-Awaji earthquake in Japan. Prev Med. 2000;31: 467-473.

[38] Matsumoto K, Yoshikawa H. Human papillomavirus infection and the risk of cervical cancer in Japan. J Obstet Gynaecol Res. 2013;39: 7-17.

[39] Nohara M. Impact of the Great East Japan Earthquake and tsunami on health, medical care and public health systems in Iwate Prefecture, Japan, 2011. Western Pac Surveill Response J. 2012;2(4):24-30.

[40] Aitsi-Selmi A, Egawa S, Sasaki H, Wannous C, Murray V. The Sendai Framework for Disaster Risk Reduction: Renewing the Global Commitment to People's Resilience, Health, and Well-being. Int J Disaster Risk Sci. 2015;6:164–176

Supporting information

S1 Table. Areas where cervical cancer screening is covered by the Miyagi Cancer Association.

S1 Figure. Change in the cervical cancer screening rate (CCS-R) in 23 areas that were screened at the hospital of a medical center in Miyagi Prefecture. The bar graph shows the CCS-R (left vertical axis; unit, %), and the line graph shows the population (right vertical axis; unit, number of people).

Figure

Click here to access/download;Figure;ScreeningFigs(revised).pptx

Fig 1

A

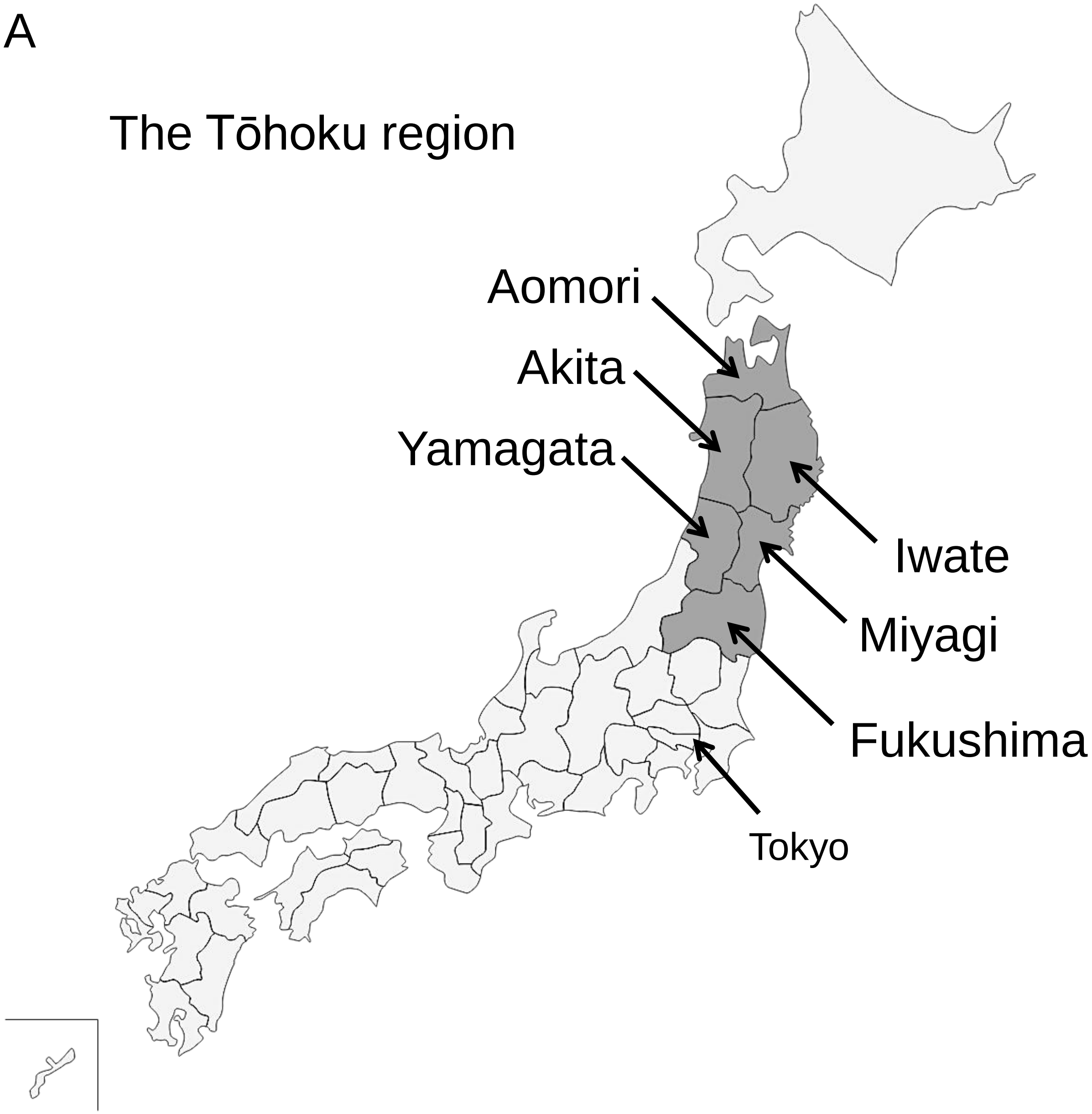

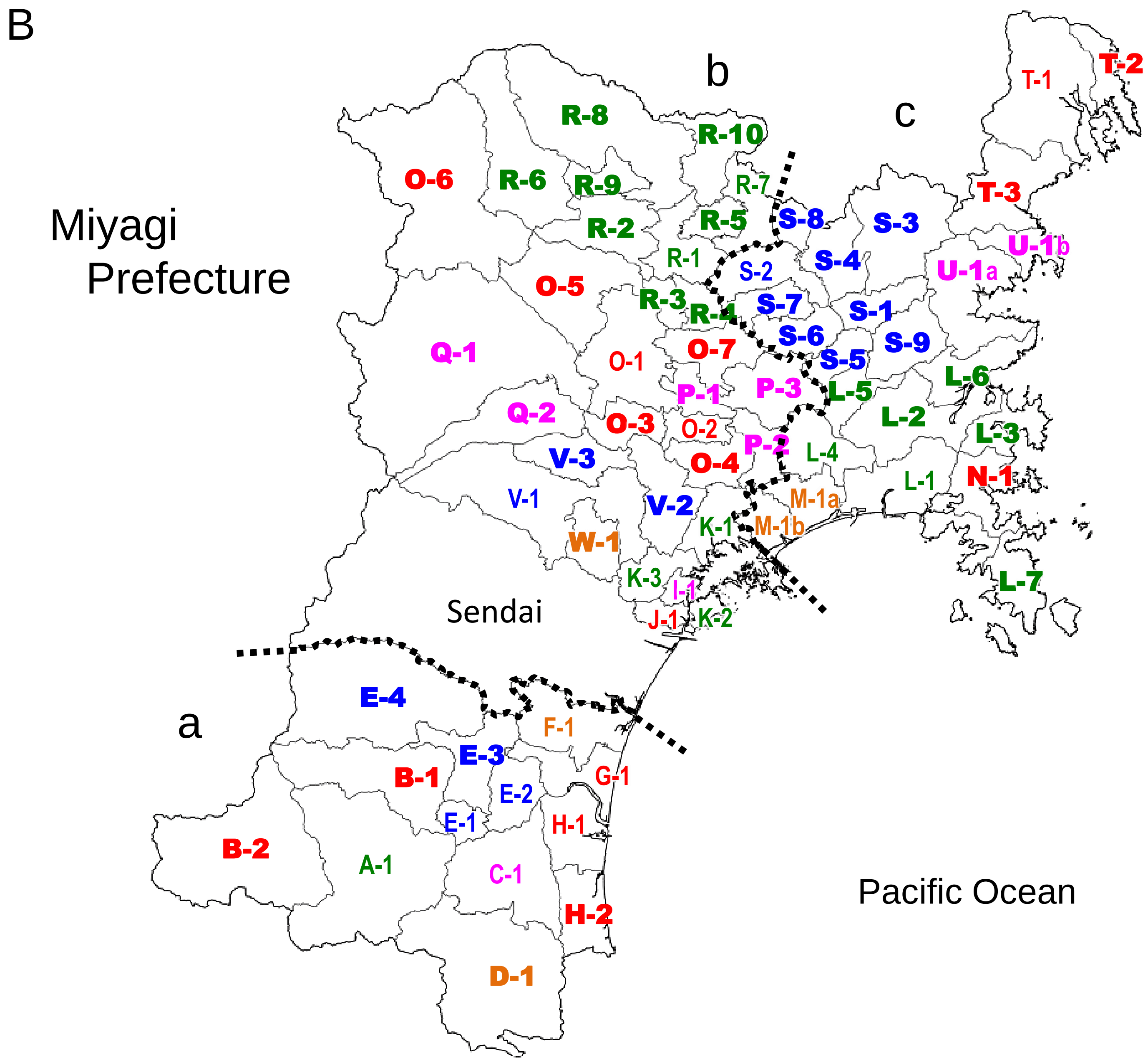
B
Miyagi
Prefecture
b
c
a
R-8
R-10
R-7
O-6
R-6
R-9
R-2
R-5
S-8
S-3
T-1
T-2
T-3
U-1b
U-1a
R-1
S-2
S-4
O-5
R-3
R-4
S-7
S-1
S-6
S-9
S-5
Q-1
O-1
O-7
P-1
P-3
L-5
L-6
L-2
Q-2
O-3
O-2
P-2
L-4
L-3
V-3
O-4
L-1
N-1
V-1
V-2
M-1a
K-1
M-1b
W-1
K-3
I-1
L-7
Sendai
J-1
K-2
E-4
F-1
E-3
B-1
G-1
E-2
E-1
H-1
B-2
A-1
C-1
H-2
D-1
Pacific Ocean

Fig 1

a

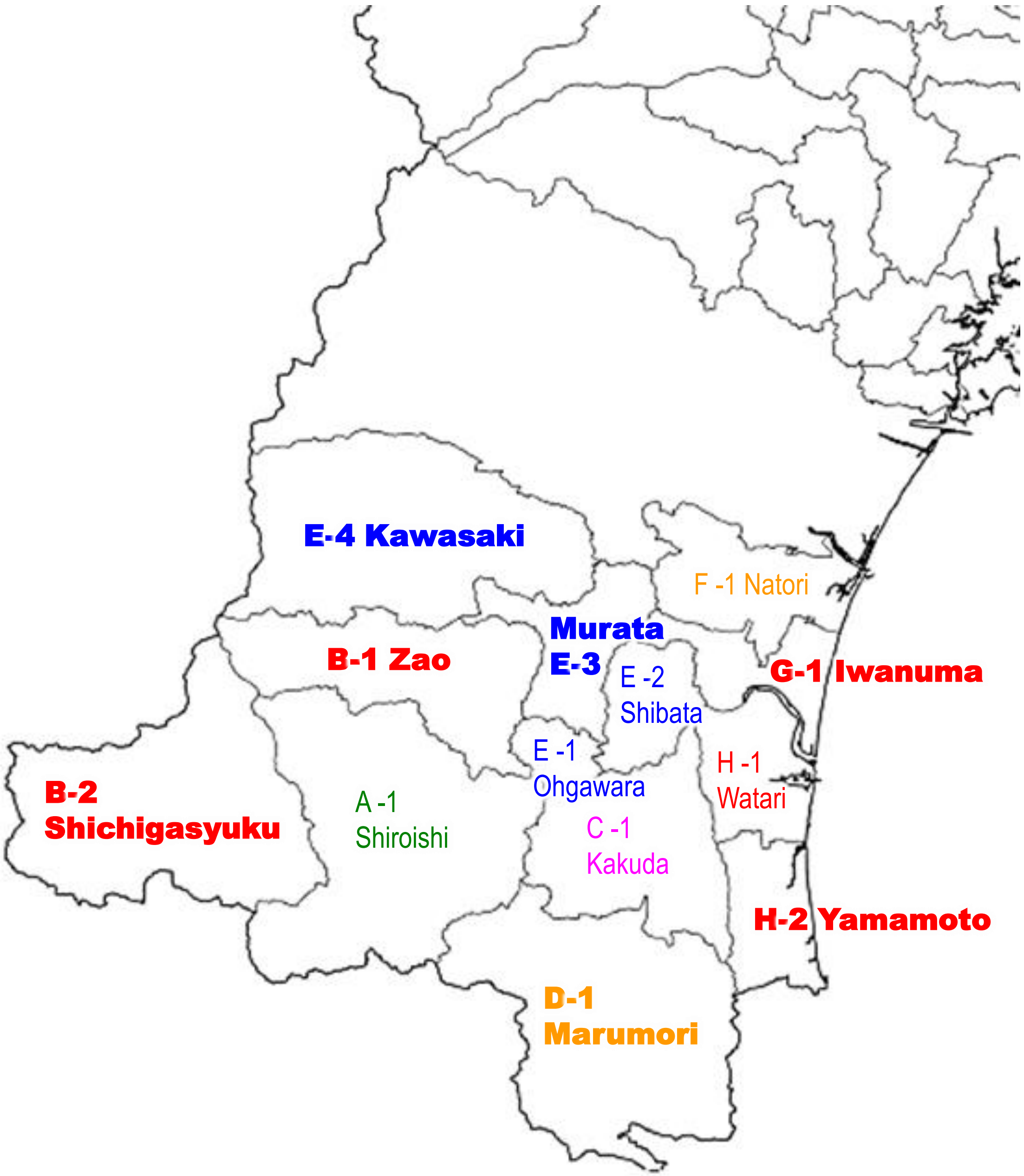
E-4 Kawasaki
F -1 Natori
Murata
E-3
B-1 Zao
G-1 Iwanuma
E -2
Shibata
E -1
Ohgawara
H -1
Watari
B-2
Shichigasyuku
A -1
Shiroishi
C -1
Kakuda
H-2 Yamamoto
D-1
Marumori

Fig 1

b

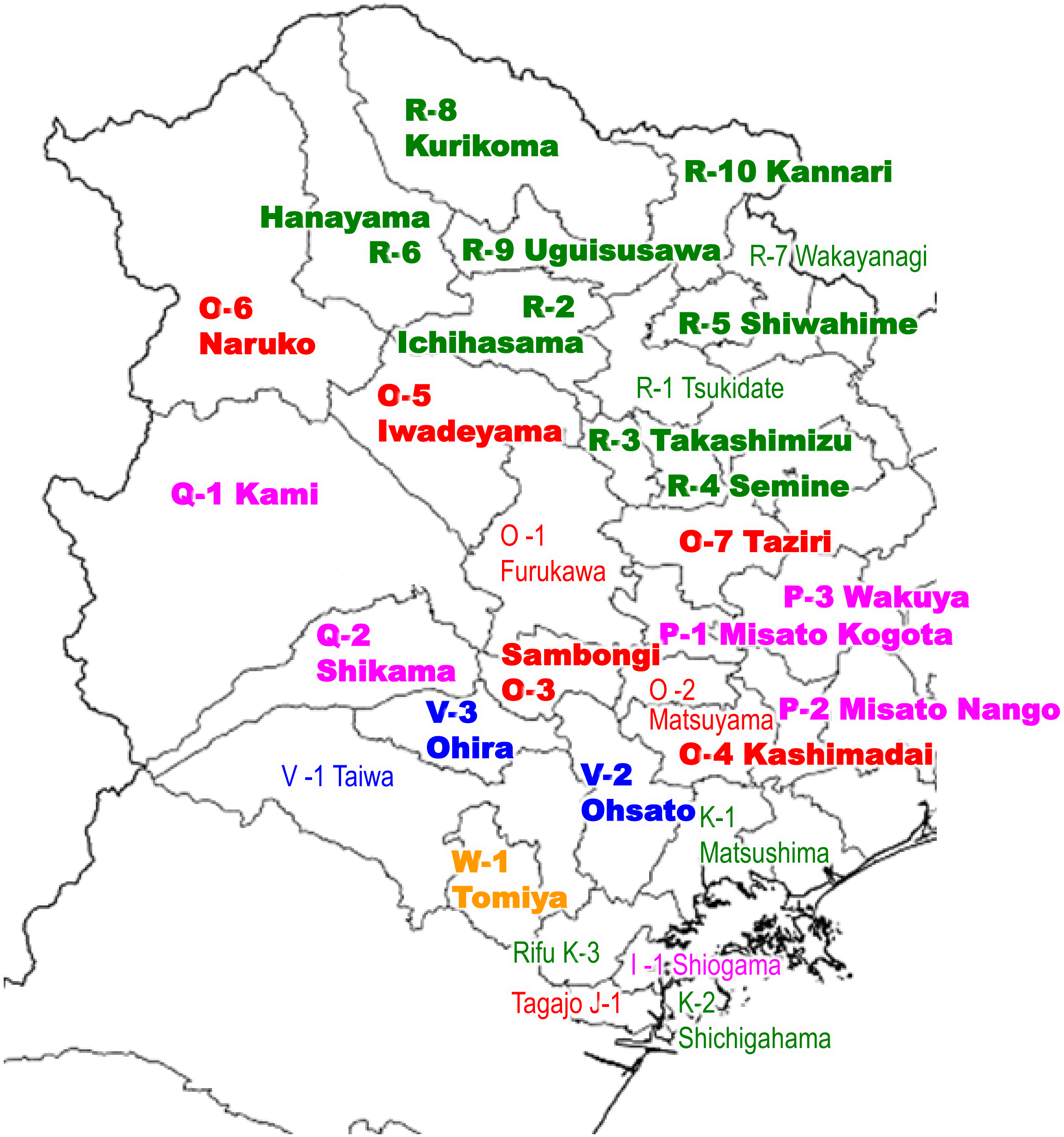
R-8
Kurikoma
R-10 Kannari
Hanayama
R-6
R-9 Uguisusawa
R-7 Wakayanagi
O-6
Naruko
R-2
Ichihasama
R-5 Shiwahime
R-1 Tsukidate
O-5
Iwadeyama
R-3 Takashimizu
R-4 Semine
Q-1 Kami
O -1
Furukawa
O-7 Taziri
P-3 Wakuya
Q-2
Shikama
P-1 Misato Kogota
Sambongi
O-3
O -2
Matsuyama
P-2 Misato Nango
V-3
Ohira
O-4 Kashimadai
V -1 Taiwa
V-2
Ohsato
K-1
Matsushima
W-1
Tomiya
Rifu K-3
I -1 Shiogama
Tagajo J-1
K-2
Shichigahama

Fig 1

c

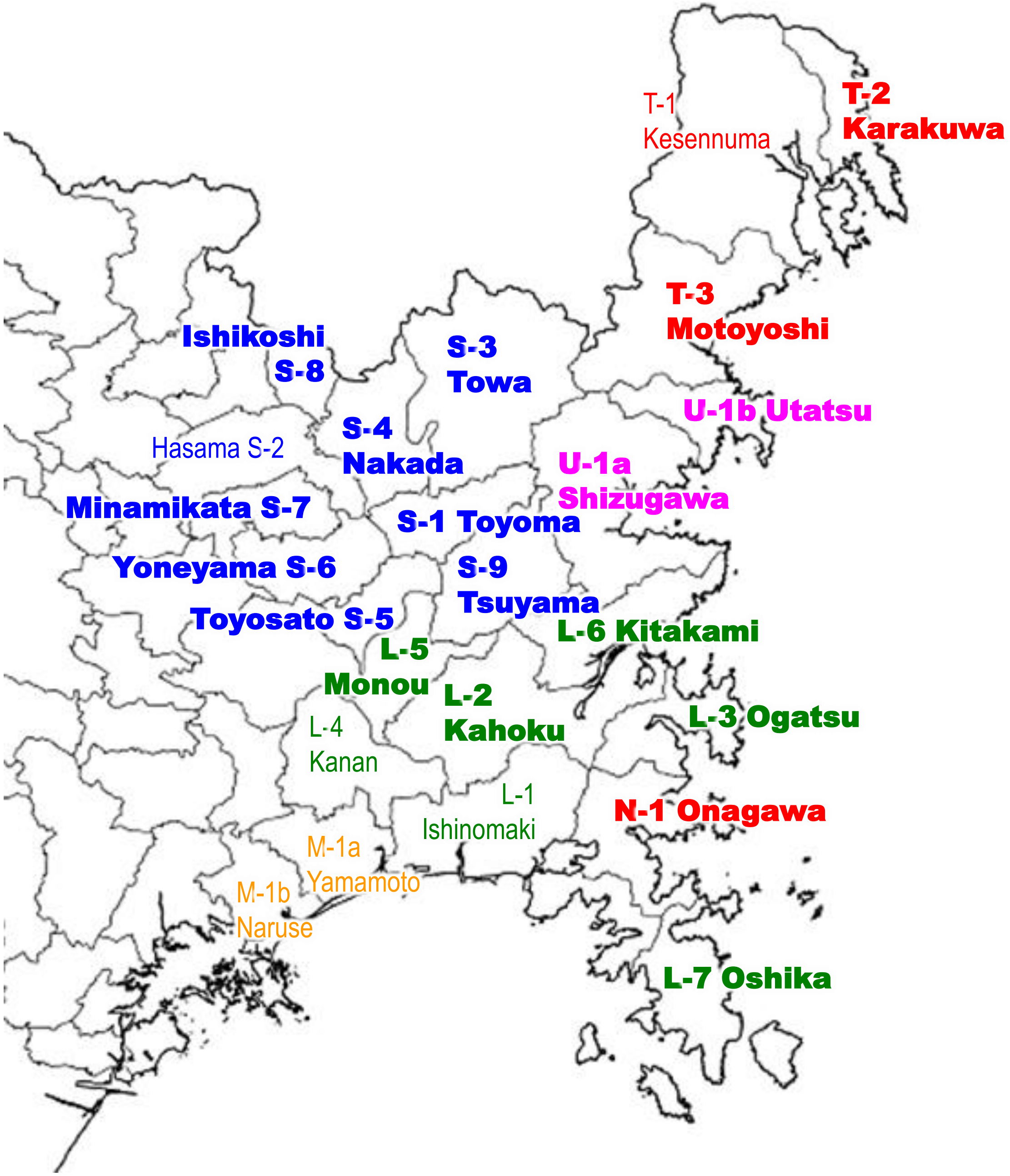

T-1
Kesennuma
T-2
Karakuwa
T-3
Motoyoshi
Ishikoshi
S-8
S-3
Towa
U-1b Utatsu
S-4
Nakada
Hasama S-2
U-1a
Shizugawa
Minamikata S-7
S-1 Toyoma
Yoneyama S-6
S-9
Tsuyama
Toyosato S-5
L-6 Kitakami
L-5
Monou
L-2
Kahoku
L-3 Ogatsu
L-4
Kanan
L-1
Ishinomaki
N-1 Onagawa
M-1a
Yamamoto
M-1b
Naruse
L-7 Oshika

Fig 2

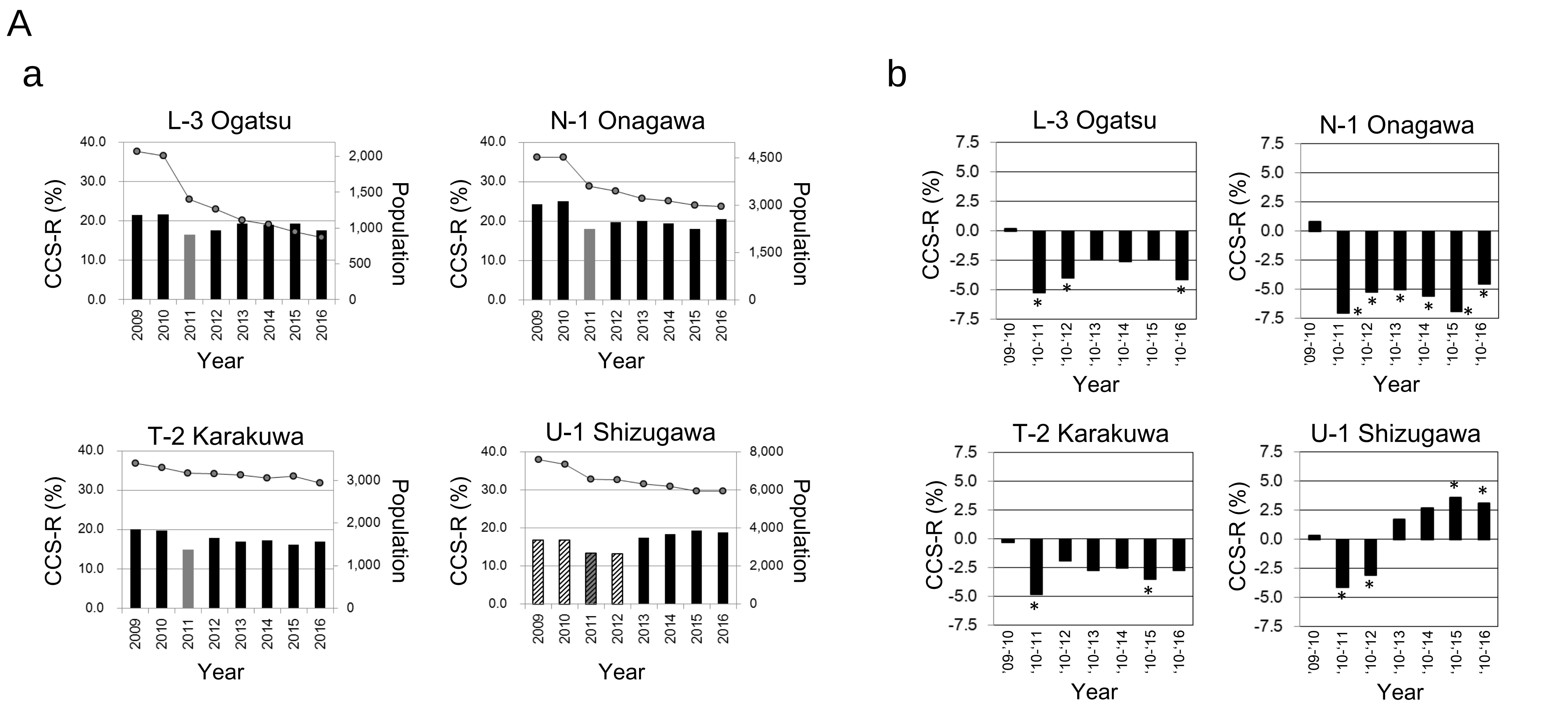
A
a
L-3 Ogatsu
N-1 Onagawa
T-2 Karakuwa
U-1 Shizugawa
CCS-R (%)
Population
Year
2009
2010
2011
2012
2013
2014
2015
2016
b
L-3 Ogatsu
N-1 Onagawa
T-2 Karakuwa
U-1 Shizugawa
CCS-R (%)
Year
'09-'10
'10-'11
'10-'12
'10-'13
'10-'14
'10-'15
'10-'16

Fig 2

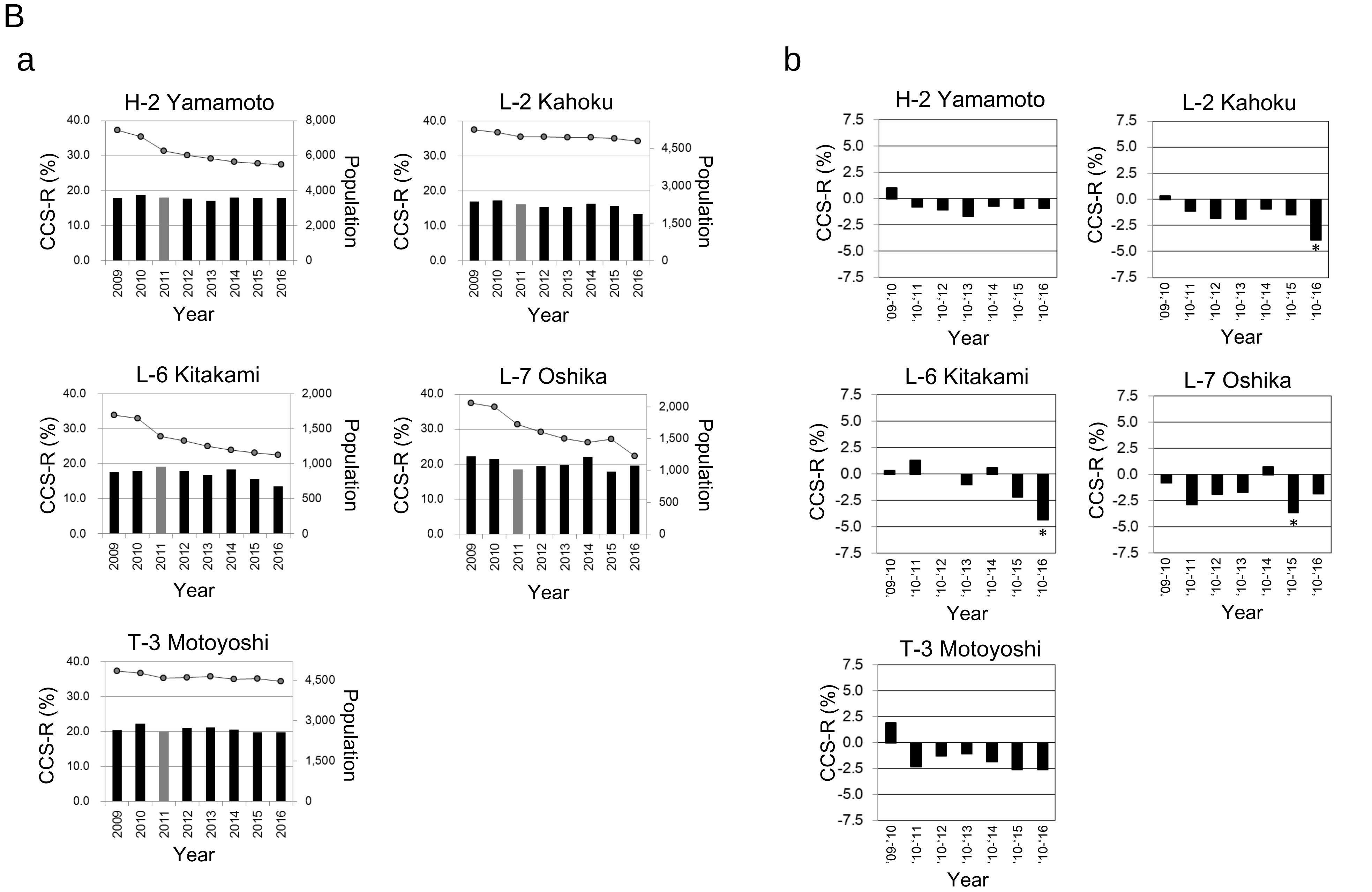
B
a
b
H-2 Yamamoto
L-2 Kahoku
L-6 Kitakami
L-7 Oshika
T-3 Motoyoshi
CCS-R (%)
Population
Year
2009
2010
2011
2012
2013
2014
2015
2016
'09-'10
'10-'11
'10-'12
'10-'13
'10-'14
'10-'15
'10-'16
*

Fig 3

A

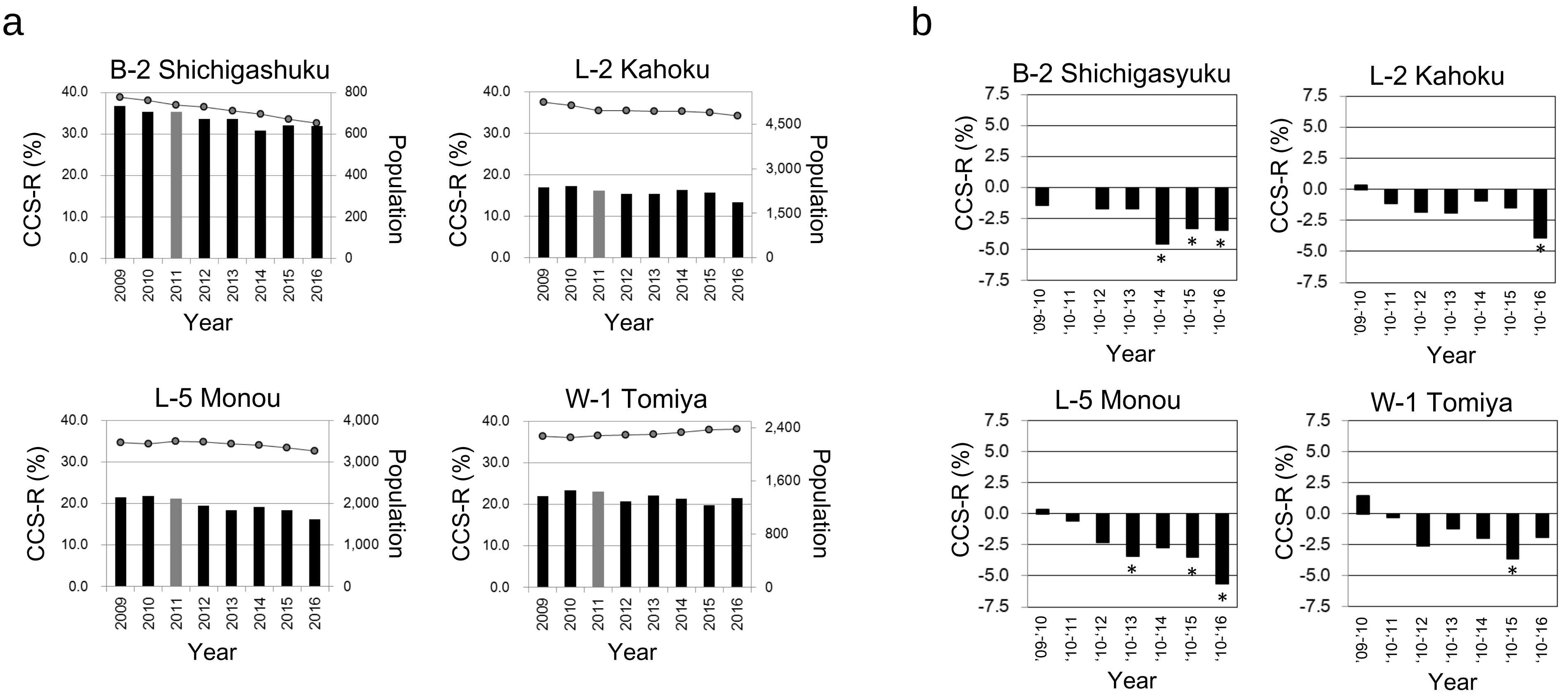
a
B-2 Shichigashuku
L-2 Kahoku
L-5 Monou
W-1 Tomiya
CCS-R (%)
Population
Year
0.0 10.0 20.0 30.0 40.0
0 200 400 600 800
0 1,500 3,000 4,500
0 1,000 2,000 3,000 4,000
0 800 1,600 2,400
2009 2010 2011 2012 2013 2014 2015 2016
b
B-2 Shichigasyuku
L-2 Kahoku
L-5 Monou
W-1 Tomiya
CCS-R (%)
Year
7.5 5.0 2.5 0.0 -2.5 -5.0 -7.5
'09-'10 '10-'11 '10-'12 '10-'13 '10-'14 '10-'15 '10-'16
*

Fig 3

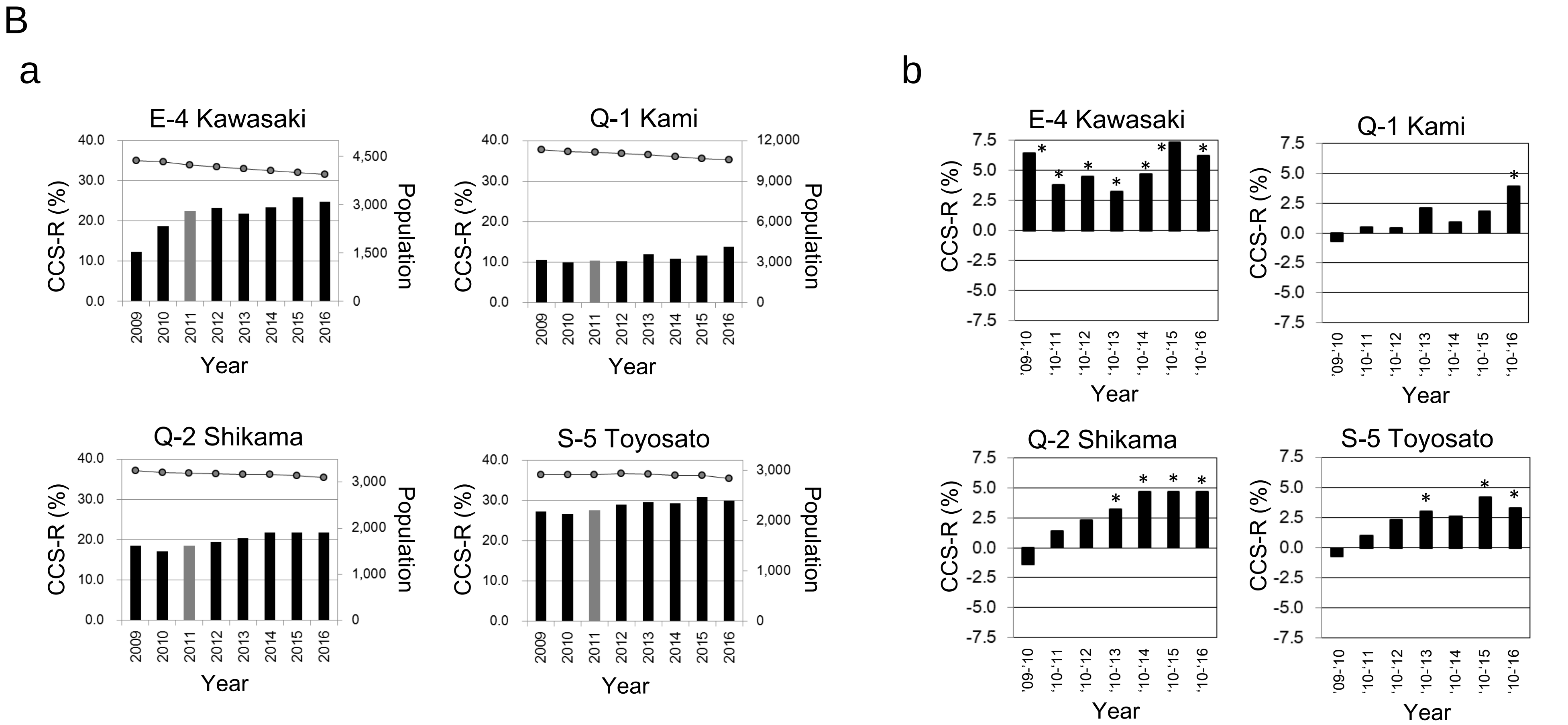
B
a
E-4 Kawasaki
Q-1 Kami
Q-2 Shikama
S-5 Toyosato
CCS-R (%)
Population
Year
2009
2010
2011
2012
2013
2014
2015
2016
b
'09-'10
'10-'11
'10-'12
'10-'13
'10-'14
'10-'15
'10-'16
*

Fig 4

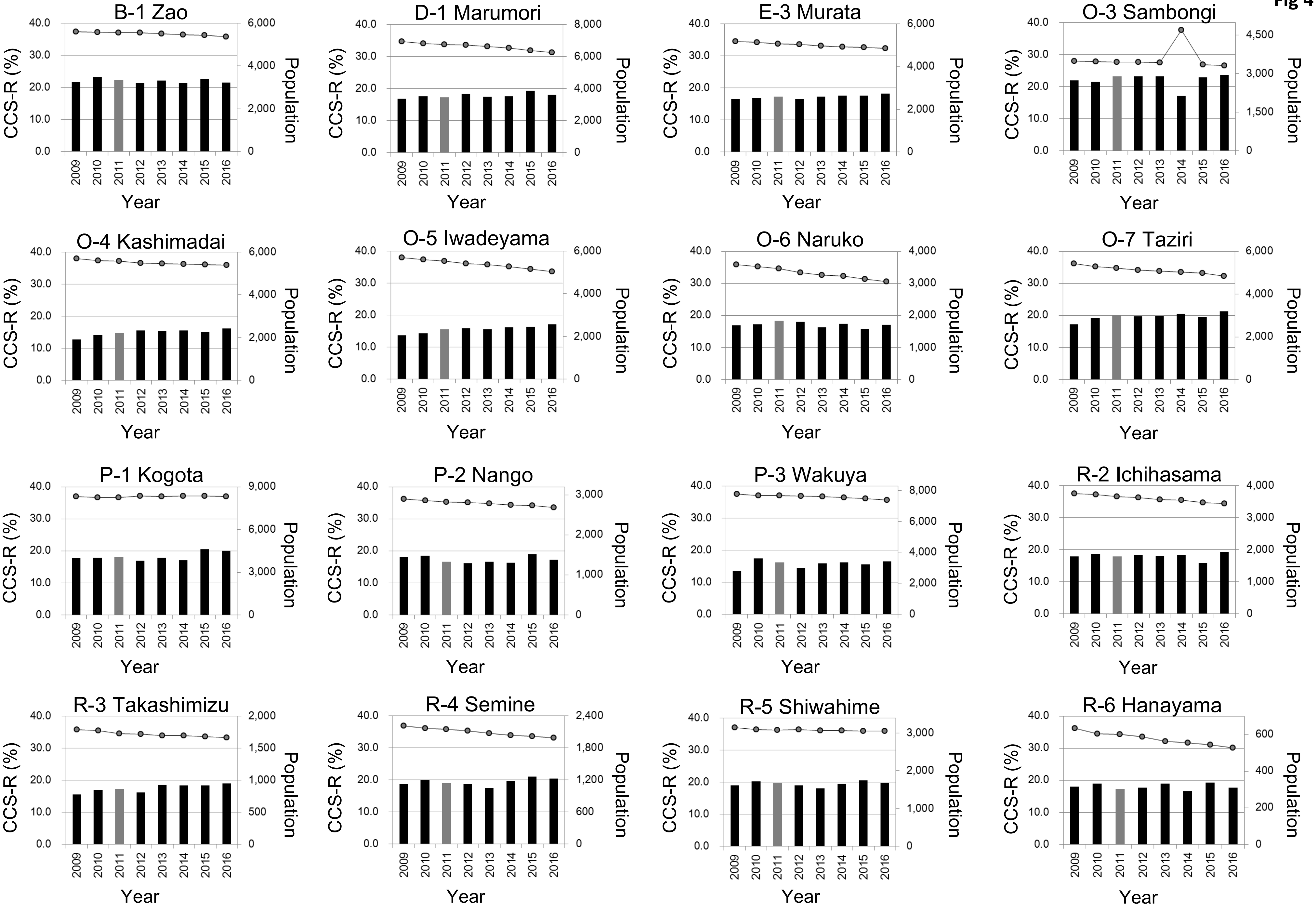
B-1 Zao
D-1 Marumori
E-3 Murata
O-3 Sambongi
O-4 Kashimadai
O-5 Iwadeyama
O-6 Naruko
O-7 Taziri
P-1 Kogota
P-2 Nango
P-3 Wakuya
R-2 Ichihasama
R-3 Takashimizu
R-4 Semine
R-5 Shiwahime
R-6 Hanayama
CCS-R (%)
Population
Year
2009
2010
2011
2012
2013
2014
2015
2016

Fig 4 cont'd

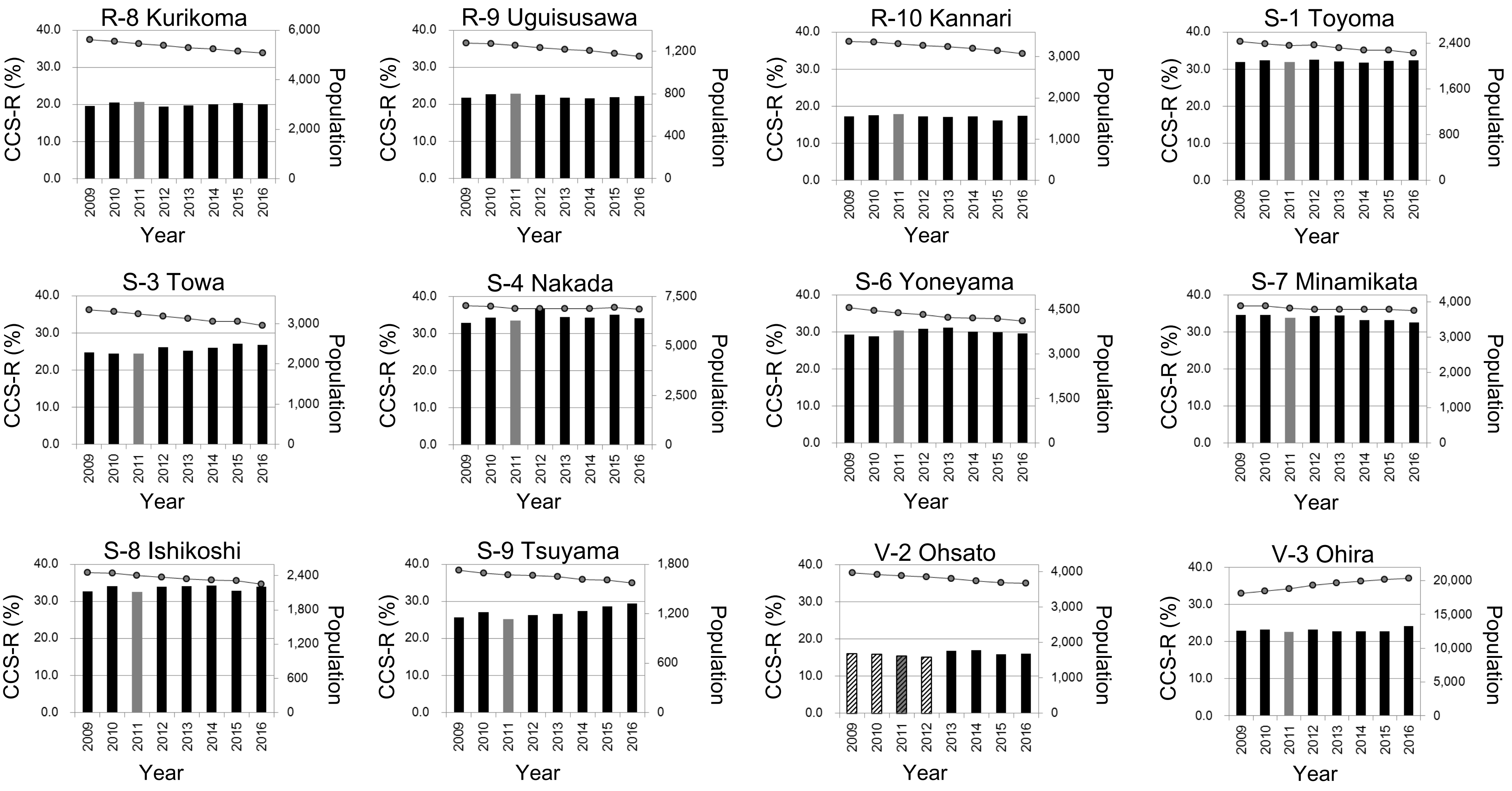
R-8 Kurikoma
R-9 Uguisusawa
R-10 Kannari
S-1 Toyoma
S-3 Towa
S-4 Nakada
S-6 Yoneyama
S-7 Minamikata
S-8 Ishikoshi
S-9 Tsuyama
V-2 Ohsato
V-3 Ohira
CCS-R (%)
Population
Year
2009
2010
2011
2012
2013
2014
2015
2016

Fig 5

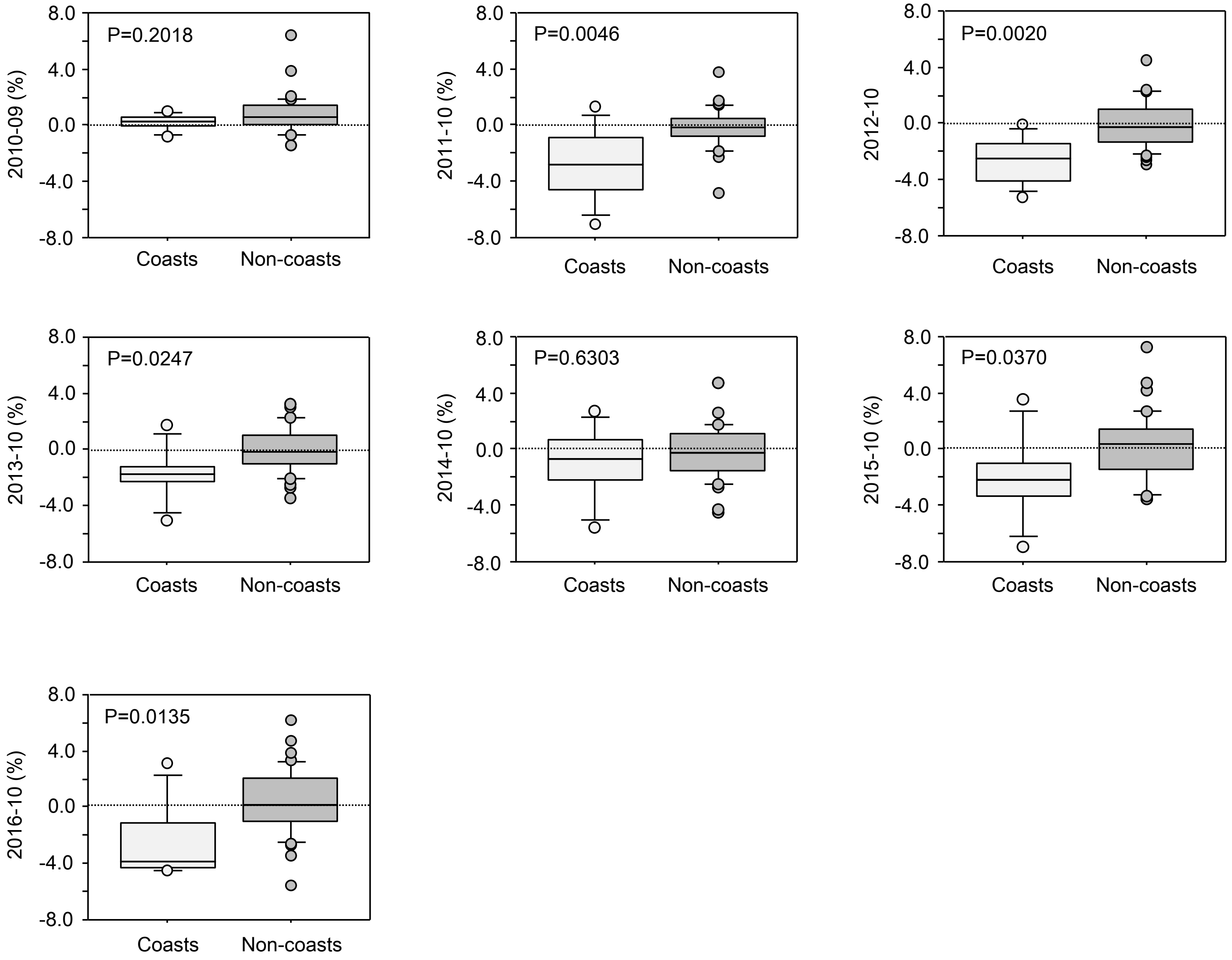

P=0.2018
2010-09 (%)
P=0.0046
2011-10 (%)
P=0.0020
2012-10
P=0.0247
2013-10 (%)
P=0.6303
2014-10 (%)
P=0.0370
2015-10 (%)
P=0.0135
2016-10 (%)
8.0
4.0
0.0
-4.0
-8.0
Coasts
Non-coasts

Click here to access/download
Supporting Information
suppl(revised).pptx

Supporting Information2

Click here to access/download
Supporting Information
S_Table(revised).xlsx

Cervical cancer screening rates before and after the Great East Japan Earthquake in the Miyagi Prefecture, Japan

Yasuhiro Miki[1], Toru Tase[2], Hideki Tokunaga[3], Nobuo Yaegashi[3], Kiyoshi Ito[1,4*]

[1] Department of Disaster Obstetrics and Gynecology, International Research Institute of Disaster Science, Tohoku University, Sendai, Japan.

[2] Cancer Detection Center, Miyagi Cancer Society, Sendai, Japan.

[3] Department of Obstetrics and Gynecology, Tohoku University Graduate School of Medicine, Sendai, Japan.

[4] Disaster Medical Science Group, Core Research Cluster of Disaster Science, Tohoku University, Sendai, Japan.

Running Title: Cervical cancer screening and the Great East Japan Earthquake

*Corresponding author:

E-mail: kito@med.tohoku.ac.jp (KI)

Abstract

After disasters, issues pertaining to women's health such as irregular periods and bleeding are well surveyed. However, the management of women's health, especially changes in the rate of health checkups, has not been investigated. In the present study, we focused on the change in the cervical cancer screening rates (CCS-Rs) before and after the Great East Japan Earthquake in Miyagi Prefecture, Japan. The earthquake had a magnitude of 9.0, a profound disaster. We examined the CCS-R from 2009 to 2016 in 45 areas of the Miyagi Prefecture. Screening was completed using mobile vans. In the 4 areas impacted by the tsunami after the earthquake, a marked decrease in the CCS-R was observed in 2011 when the earthquake took place (more than a 3% decrease compared with that in the previous year). The CCS-Rs in these 4 regions remained lower in 2016 than in the previous year. In 2009–2016 except for 2014, CCS-Rs in coastal areas (9 areas) were significantly lower than those in the non-coastal areas (36 areas). A delay in seeking healthcare, also known as "patient's delay," is considered as one of the problems of cancer treatment in affected areas. It is possible that a decrease in the CCS-R may lead to low detection of advanced stages of cancer. Therefore, the establishment of a comprehensive medical system including medical screening after a disaster is important for the management of women's health.

Introduction

The cervical cancer incidence in Japan is 14.7 (age-standardized rates per 100,000 population), which is higher than that in other developed countries such as United States (6.5), Australia (6.0), and South Korea (8.4) and similar to that in India (14.7) and the Philippines (14.9) (Data from Cancer Today - IARC, https://gco.iarc.fr/today/online-analysis-table, accessed 2020.1.17). Furthermore, vaccination coverage in Japan (under 1%) is significantly lower than that in other countries where cervical cancer vaccines are licensed [1,2]. Therefore, cervical cancer screening is considered crucial. However, the screening rate in Japan is considerably lower than that in Western and other countries [3]. It is reported that the screening rates in the United States and Britain and those in France and South Korea are approximately 80% and 70%, respectively [3]. Furthermore, the screening rate in Japan (33.7% [age group: 20-69 years]) is lower than that in Australia (56%) [3,4,5]. An increase in the coverage of women receiving periodic cervical cancer screening is urgently needed in Japan. The rate of cervical cancer screening in the Miyagi Prefecture (42.1%; age group 20-69 years) was the second highest among the 47 prefectures in Japan in 2016 [5]. In the Miyagi Prefecture, the mobile van service (or mobile examination unit) offering cervical cancer screening serves rural areas where it is difficult to obtain an examination

in a hospital or medical center [6,7]. There are two types of cancer screenings: population-based screening and opportunistic screening such as private and company medical check-ups. All of the mobile van screenings are population-based screenings.

On March 11, 2011, a magnitude 9.0 earthquake, termed the Great East Japan Earthquake, struck the east area of Japan. The abbreviation “3.11” has been used for this earthquake, mainly by the Japanese mass media as the earthquake occurred on March 11, 2011. After the Great East Japan Earthquake, higher-than-expected tsunami waves hit coastal zones of Tohoku and caused devastating damage. The economic loss was serious, and the damage caused by the earthquake was estimated to be approximately 16–25 trillion yen [8]. The tsunami-impacted area was predicted to require 10 years for reconstruction [9]. In the Miyagi Prefecture, a mobile van service offering cervical cancer screening covered the areas severely affected by the tsunami after the Great East Japan Earthquake. The screening resumed promptly to maintain the health of the residents in the disaster area. In many areas, cervical cancer screening was resumed in April of the year of the disaster, 2011. However, in coastal areas (L-2, L-3, T-3, U-1b in Fig. 1), the re-initiation of screenings was delayed from July to December, 2011. The screening was resumed only in Oshika (L-7 in Fig. 1) in February, 2012 following the earthquake. Unfortunately, the people affected by the disaster continue to be mentally

and financially unstable and cannot afford to maintain their health [10,11,12].

It is well known that conflicts and disasters have a major impact on healthcare and result in delays in the diagnosis and treatment of patients with cancer [10,11]. Furthermore, the social isolation caused by a disaster may delay the opportunity to examine and treat patients with cancer [12]. A study at a city hospital in 2005 in New Orleans, affected by Hurricane Katrina, found that the mean time from last cytology (Pap test) to diagnosis of cervical cancer was significantly longer after the hurricane (7.7 years) than before the hurricane (4.2 years) (Abstract of the 2001 Western Association of Gynecologic Oncologists Annual Meeting, doi: 10.1016/j.ygyno.2011.07.082 [13]). In addition, the clinical stage of cervical cancer at diagnosis was significantly higher after the hurricane than before the hurricane [13]. Women's health hazards such as abnormal menstrual cycles, pelvic inflammation, and lower genital tract infections after disasters also have been reported in various countries [14-18]. Although the failure of health management is considered to lead to long-term health risks, the cervical cancer screening rate (CCS-R) after a disaster remains unexamined. In this study, we examined the trend of CCS-Rs from the Japan fiscal year (April 1 to March 31) 2009–2016 in 45 areas of the Miyagi Prefecture where cervical screenings were performed by the Miyagi Cancer Society (Fig 1, S1 Table).

Fig 1. The Tohoku region in Japan and the survey area in the Miyagi Prefecture. (A) The Tohoku Region has six prefectures: Aomori Prefecture, Iwate Prefecture, Miyagi Prefecture, Akita Prefecture, Yamagata Prefecture, and Fukushima Prefecture. (B) The survey area in Miyagi. Enlarged views are presented for each of the areas a, b, and c. Also, see S1 Table for details. Bold style code numbers represent areas where cervical cancer screenings were performed by mobile vans.

Materials and methods

CCS-Rs were obtained from the Annual Report in 2009–2016 and were compiled by the Miyagi Cancer Society (Sendai, Miyagi, Japan). The Miyagi Cancer Society was established as a public interest incorporated foundation and has formed a cancer screening and cancer registration business in Miyagi Prefecture, Japan. The annual report is also compiled as one of the functions of the Miyagi Cancer Society. The population of each region in each year was also extracted from this Annual Report.

The incidence of cervical cancer has increased in Japanese women in their mid-20s [19]. Therefore, Japanese cervical cancer screening guidelines recommend to

begin screening at the age of 20 years [20]. The CCS-R was defined by the following equation: CCS-R = {(Number of women aged 20 years and older who underwent cervical cancer screening) ÷ (Total population of women aged 20 years and older)} × 100 (%). The difference in CCS-R for each year compared with the year before the Great East Japan Earthquake was calculated as follows: Δ[CCS-R in the year] = {CCS-R in the year (%)} − {CCS-R in the year before the Great East Japan Earthquake, 2010 (%)}; *e.g.*, Δ[CCS-R 2011] (%) = CCS-R in 2011 (%) − CCS-R in 2010 (%). The difference in population in each area was calculated similarly: Δ [Pop. in the year] = 100 (%) − {(Pop. in the year) ÷ (Pop. in the year before the earthquake, 2010) × 100 (%)}.

In the Miyagi Prefecture, cervical cancer screening was performed in the mobile van or the medical center/hospital. The medical exam statistics at the medical center or hospitals in the Miyagi Prefecture included a large number of sporadic outpatients, who complained of symptoms such as irregular bleeding. Therefore, the results of screening at medical facilities (23 areas) are shown in S1 Figure. The study area (45 areas) is shown in Figure 1 and S1 Table. In those regions, cervical cancer screening was provided by a mobile van. All of the data were processed using Microsoft Excel (Microsoft Corporation, Redmond, WA, USA). Mann–Whitney U test with StatView ver. 5.0 (SAS Institute Inc., San Francisco, CA, USA) was used for comparison between the

groups. This research was approved by the ethics committees of the International Research Institute of Disaster Science, Tohoku University (Sendai, Japan) and Miyagi Cancer Society (Sendai, Japan).

Results

Differences in the CCS-R in each region from 2010 to 2016 are summarized in Table 1. Among the areas covered by the mobile van, the Δ[CCS-R 2011] in the 4 regions markedly decreased (over −3.0%) (Fig 2A): Ogatsu −5.2% (L-3), Onagawa −7.0% (N-1), Karakuwa −4.8% (T-2), and Shizugawa −4.1% (U-1a). These four coastal regions were affected by the tsunami. In Ogatsu (L-3), the CCS-Rs tended to recover the year after the earthquake (2013) and decreased approximately by 2.5% in 2013–2015 compared with the rate one year prior to the earthquake (2010). However, the Δ[CCS-R 2016] was −4.1% (Fig 2Ab). The population from 2011 (Δ[Pop.2011]) to 2016 (Δ[Pop.2016]) in Ogatsu (L-3) remained at −30.3% to −56.8% (Fig 2Aa). In Onagawa (N-1), where the decrease in the CCS-R was the highest in the year the earthquake occurred (Δ[CCS-R 2011], −7.0%), a high reduction ratio (−5.2 → −5.0 → −5.6 → −6.9 → −4.5) was observed even after the earthquake from (Δ[CCS-R 2012] to Δ[CCS-R

2016]) (Fig 2Ab). The Δ[Pop.2011] to Δ[Pop.2016] in Onagawa (N-1) remained at −20.6% to −34.8% (Fig 2Aa). In Karakuwa (T-2), although the CCS-R recovered to a decreased rate of less than 2.0% (Δ[CCS-R 2012]), it decreased by 3.0% from (Δ[CCS-R 2013] to Δ[CCS-R 2016]) (Fig 2Ab). The Δ[Pop.2011] to Δ[Pop.2016] in Karakuwa (T-2) remained at −4.0% to −11.1% (Fig 2Aa). In Shizugawa (U-1a), the CCS-R decreased by 3% in the year following the earthquake (Δ[CCS-R 2012]) but recovered to the level one year before the earthquake (2010) in 2013 after the merger with Utatsu (U-1b) and increased by more than 3.0% in 2015 and 2016 (Table 1). The Δ[Pop.2011] to Δ[Pop.2016] in Minamisanriku (U-1 = U-1a + U-1b) remained at −10.5% to −19.1% (Table 1).

Table 1. Differences in the Cervical Cancer Screening Rates Between Before and After the Great East Japan Earthquake.

**Code No.**			**2010–2009**	**2011–2010**	**2012–2010**	**2013–2010**	**2014–2010**	**2015–2010**	**2016–2010**
B	1	Zao Town	1.5	−0.8	−1.8	−1.0	−1.8	−0.6	−1.7
	2	Shichigasyuku Town	−1.4	0.0	−1.7	−1.7	***−4.5***	***−3.3***	***−3.4***

D	1	Marumori Town	0.7	−0.2	0.9	−0.1	0.1	1.8	0.5
E	3	Murata Town	0.4	0.5	−0.4	0.5	0.7	0.8	1.4
	4	Kawasaki Town	***6.4***	***3.8***	***4.5***	***3.2***	***4.7***	***7.3***	***6.2***
H	2	Yamamoto	1.0	−0.8	−1.1	−1.7	−0.7	−0.9	−0.9
L	2	Kahoku	0.3	−1.1	−1.8	−1.9	−0.9	−1.5	***−3.9***
	3	Ogatsu	0.2	***−5.2***	***−4.0***	−2.4	−2.6	−2.4	***−4.1***
	5	Monou	0.3	−0.6	−2.3	***−3.4***	−2.7	***−3.5***	***−5.6***
	6	Kitakami	0.3	1.3	0.0	−1.0	0.6	−2.2	***−4.3***
	7	Oshika	−0.8	−2.9	−1.9	−1.7	0.7	***−3.6***	−1.8
N	1	Onagawa Town	0.8	***−7.0***	***−5.2***	***−5.0***	***−5.6***	***−6.9***	***−4.5***
O	3	Sambongi	−0.6	1.7	1.7	1.7	***−4.3***	1.4	2.2
	4	Kashimadai*	1.4	0.5	1.4	1.1	1.3	0.8	2.0
	5	Iwadeyama†	0.6	1.3	1.6	1.3	1.8	2.0	2.8
	6	Naruko	0.2	1.1	0.8	−0.9	0.2	−1.4	−0.1
	7	Taziri	2.1	0.9	0.5	0.6	1.2	0.3	2.0
P	1	Misato Town	0.2	0.1	−1.0	0.0	−0.8	2.6	2.1

		Kogota							
	2	Misato Town Nango	0.5	−1.9	−2.4	−1.8	−2.2	0.5	−1.3
	3	Wakuya Town‡	***3.9***	−1.2	−2.9	−1.5	−1.2	−1.9	−0.9
Q	1	Kami Town	−0.7	0.5	0.4	2.1	0.9	1.8	***3.9***
	2	Shikama Town†	−1.4	1.4	2.3	***3.2***	***4.7***	***4.7***	***4.7***
R	2	Ichihasama	0.8	−0.9	−0.4	−0.6	−0.4	−2.9	0.6
	3	Takashimizu	1.4	0.3	−0.8	1.5	1.4	1.4	2.0
	4	Semine	1.3	−1.0	−1.3	−2.5	−0.3	1.1	0.5
	5	Shiwahime	1.3	−0.5	−1.3	−2.1	−0.8	0.4	−0.4
	6	Hanayama	0.9	−1.7	−1.2	0.0	−2.3	0.4	−1.2
	8	Kurikoma	1.0	0.1	−1.1	−0.8	−0.6	−0.2	−0.6
	9	Uguisusawa	1.0	0.1	−0.2	−1.0	−1.1	−0.8	−0.5
	10	Kannari	0.3	0.3	−0.3	−0.4	−0.2	−1.4	−0.1
S	1	Toyoma	0.4	−0.4	0.2	−0.2	−0.5	−0.1	0.1
	3	Towa	−0.3	0.0	1.8	0.8	1.6	2.7	2.3

	4	Nakada	1.3	−0.7	2.4	0.2	0.1	0.8	−0.1
	5	Toyosato	−0.7	1.0	2.3	***3.0***	2.6	***4.2***	***3.3***
	6	Yoneyama	−0.5	1.5	2.0	2.3	1.3	1.1	0.8
	7	Minamikata	0.1	−0.8	−0.3	−0.2	−1.4	−1.4	−2.0
	8	Ishikoshi	1.4	−1.6	−0.2	0.0	0.2	−1.2	−0.1
	9	Tsuyama	1.5	−1.9	−0.8	−0.5	0.3	1.6	2.3
T	2	Karakuwa	−0.3	***−4.8***	−1.9	−2.7	−2.5	***−3.5***	−2.7
	3	Motoyoshi	1.9	−2.3	−1.3	−1.1	−1.8	−2.6	−2.6
U	1a	Shizugawa	0.3	***−4.1***	***−3.1***	1.7	2.7	***3.6***	***3.1***
	1b	Utatsu	−0.4	−2.7	***−4.1***	–	–	–	–
V	2	Ohsato Town	−0.2	−0.4	−0.7	1.0	1.1	0.0	0.2
	3	Ohira Village	0.2	−0.6	0.0	−0.4	−0.4	−0.4	1.0
W	1	Tomiya City	1.4	−0.3	−2.6	−1.2	−2.0	***−3.6***	−1.9

Areas covered by mobile vans were examined. Screening by the mobile vans *since 2015, †since 2013, and ‡since 2011; Bold italic, change of 3% or more; U-1, Shizugawa (U-1a) and Utatsu (U-1b) were integrated in 2013. The 2010 cervical cancer screening rate was compared with that of 2009.

Fig 2. Change in the CCS-R in coastal areas.

(A) a. Coastal areas showing a remarkable decrease in the cervical cancer screening rates (CCS-Rs) in the year following the Great East Japan Earthquake (over −3.0%). The bar graph shows the CCS-R (%), and the line graph shows the population (number of people). b. Differences in the CCS-R between before and after the Great East Japan Earthquake in each area demonstrated in “a”. *, change of 3% or more. U-1 since Shizugawa (U-1a) and Utatsu (U-1b) were integrated in 2013; the data for 2009–2012 include a total of two regions.

(B) a. Change in CCS-R in other coastal areas. The bar graph shows the CCS-R (%), and the line graph shows the population (number of people). b. Differences in the CCS-R between before and after the Great East Japan Earthquake in each area demonstrated in “a”. *, change of 3% or more.

In the tsunami-affected coastal areas other than the above four towns, the Δ[CCS-R 2011] was as follows (Fig 2B): Yamamoto−0.8% (H-2), Kahoku −1.1% (L-2), Kitakami +1.3% (L-6), Oshika −2.9% (L-7), and Motoyoshi −2.3% (T-3) (Fig 2Bab). The Δ [Pop. 2011] was as follows; Yamamoto −11.3% to −22.4% (H-2), Kahoku −3.5%

to −7.0 (L-2), Kitakami −15.3% to −31.7% (L-6), Oshika −14.0% to −38.7% (L-7), and Motoyoshi −3.7% to −6.4% (T-3) (Fig 2Ba).

In non-coastal areas, the CCS-R declined by more than 3.0% compared with that of the year before the earthquake during the survey period in four places [Shichigashuku (B-2), Kahoku (L-2), Monou (L5), Tomiya (W-1)] (Fig 3A). In contrast, in the 4 non-coastal regions, the CCS-R increased by more than 3.0% during the survey period compared with that before the earthquake year [Kawasaki (E-4), Kami (Q-1), Shikama (Q-2), Tomiya (S-5)] (Fig 3B). No notable increase or decrease (±3.0%) was observed in other non-coastal areas (Fig 4).

Fig 3. Change in the cervical cancer screening rate (CCS-R) in non-coastal areas examined in this study.

(A) a. The CCS-R declined by more than 3.0% compared with that of the year before the earthquake (over −3.0%). The bar graph shows the CCS-R (%), and the line graph shows the population (number of people). b. Differences in the CCS-R between before and after the Great East Japan Earthquake in each area demonstrated in “a”. *, change of 3% or more.

(B) a. The CCS-R declined by more than 3.0% compared with that of the year before

the earthquake (over +3.0%). The bar graph shows the CCS-R (%), and the line graph shows the population (number of people). b. Differences in the CCS-R between before and after the Great East Japan Earthquake in each area demonstrated in “a”. *, change of 3% or more.

Fig 4. Change in the cervical cancer screening rate (CCS-R) in other non-coastal areas examined in this study.

The bar graph shows the CCS-R (%), and the line graph shows the population (number of people).

When the areas examined were divided into coastal and non-coastal areas, the CSS-R was significantly lower in the coastal areas during 2011 ($p = 0.0046$), 2012 ($p = 0.0020$), 2013 ($p = 0.0247$), 2015 ($p = 0.0370$), and 2016 ($p = 0.0135$) (Fig 5). No significant difference was found in the 2-year comparison before the earthquake (2010–2009, $p = 0.2018$) and 2014 (2014–2010, $p = 0.6303$) (Fig 5).

Fig 5. Comparison of the change in the cervical cancer screening rate in coastal

and non-coastal regions.

The bar graph shows the median value of differences in the CCS-R between before and after the Great East Japan Earthquake (%). Coast, nine coastal regions; Non-coasts, 36 non-coastal regions.

Discussion

The health of women, especially pregnant women, has been investigated in the areas affected by the Great East Japan Earthquake [21-23]; however, no research has been conducted with regard to cancer screening. In the present study, we first clarified the difference in the CCS-R of the Miyagi Prefecture before and after the Great East Japan Earthquake. In the present study, after the Great East Japan Earthquake, the CCS-R significantly decreased in the coastal areas compared with that in other areas. The coastal areas in the Miyagi Prefecture were severely impacted by an unforeseen tsunami. Even within the screening areas, a drastic population decline was observed immediately after the earthquake. It is well known that the tsunami resulted in severe human and residential damage as a result of the Great East Japan Earthquake [24,25]. Therefore, coastal populations are considered to have decreased both due to death as

well as migration as a consequence of the tsunami. After the Great East Japan Earthquake, the population also decreased in areas with a severe decrease in the CCS-R. Although the decrease in the CCS-R could have been attributed to the decline in the population, there were many areas in which there was no clear relationship between the decrease in population and the CCS-R. In the present study, we used the total population of each region for evaluation. We found that further studies that are corrected for the population of women aged over 20 years are required. The economic loss caused by the Great East Japan Earthquake in the Miyagi Prefecture was more severe in coastal areas than in inland areas (4.9 vs 1.6 trillion yen) in 2012 [24]. In coastal areas, the damage to livelihoods and social infrastructure resulted in a loss of 2.0 trillion yen and the loss due to residential damage was 1.4 trillion yen. This was 2.4- and 36.2-times higher than that in inland areas, respectively [24]. Furthermore, reconstruction in the Miyagi Prefecture was reported to be markedly lower in coastal areas than in inland areas in 2012 to 2016 [25].

From the year after the Great East Japan Earthquake to 2016, there were some areas where the CCS-R recovered; however, other areas did not show such recovery. Furthermore, in many areas where cervical cancer screening had been conducted at medical facilities by the Miyagi Cancer Society, a decline in the CCS-R was observed in

the year following the earthquake (S1 Figure). In an evidence review by CPSTF (USA), small media sources such as newspapers and educational videos as well as solicitation by telephone or letter are recommended as interventions to increase the CCS-R (https://www.thecommunityguide.org/content/task-force-findings-cancer-prevention-and-control; accessed 2020.1.17). In Japan, it was also reported that local government initiatives and education contributed to increasing the screening rate of young women [26]. However, a detailed investigation is required in areas where these screening rates have declined and awareness attempts by local government and other organizations may have been delayed. It is important for future post-disaster measures to clarify the details of the recruitment of cervical cancer screening programs for women in each area.

It is well known that conflicts and disasters have a major influence on healthcare and cause delays in diagnosis and treatment of patients with cancer [10]. In the Fukushima Prefecture, which was impacted by a triple disaster (an earthquake, a tsunami, and a nuclear accident), delays in symptom recognition to the first medical examination in patients with breast cancer have been reported [11]. The trend of risk due to patients’ delay continues even following the disaster [11]. It is also reported that social isolation due to the triple disaster in Fukushima may have delayed opportunities for medical consultation and appropriate therapy among patients with colon cancer [12].

A survey of 366 patients with cervical cancer from urban hospitals in New Orleans, USA affected by Hurricane Katrina in 2005 was conducted [13]. The mean time from last cytology (Pap test) to diagnosis of cervical cancer was significantly longer after the hurricane (7.7 years) than before the hurricane (4.2 years) [13]. In addition, the cancer stage was significantly higher ($p < 0.01$) after the hurricane (From 2006 to 2010: stage I, 40 cases; stage II, 36 cases; stage III, 32 cases; stage IV, 32 cases.) than before the hurricane (From 2000 to 2005: stage I, 101 cases; stage II, 60 cases; stage III, 37 cases; stage IV, 28 cases) [13]. The five-year overall survival rates for cervical cancer stages I and II are known to be approximately 90% and 75%, respectively [19]. Early detection by periodic screening is critical in order to prevent death from cervical cancer [20]. If regular screening is skipped as a result of the earthquake, it may be detected later as advanced cancer. This situation was found in the area that was impacted by Hurricane Katrina [13]. These reports illustrate the importance of prompt reconstruction of healthcare systems, including screening, for early detection [13,27]. Compared to the USA CCS-R, the Japan CCS-R is too low [3,5]. Therefore, it would be difficult to perform the same analysis as that in US research due to the small number of comparative patients. Otherwise, in Japan, cervical cancer screening is expected to reduce cervical cancer deaths (recommendation grade B), with evidence levels of 2++

for conventional and 2+ for liquid-based cytology [20]. There are limitations to conducting a further detailed analysis in the areas examined in this study; however, a reduction in CCS-Rs suggests that women's health will be significantly affected.

The trauma experienced during tsunamis remains a strong psychological stressor among disaster victims [28-32]. A survey after the Great East Japan Earthquake reported that difficulty settling back into daily living, the presence of preexisting illnesses, and the disruption of social networks were the most important risk factors for mental health disorders. These conditions did not improve even 2 years after the disaster [33]. Therefore, it is possible that women in the affected areas will continue to be exposed to long-term distress. Psychological stress is believed to exhibit immunosuppressive effects through the inhibition of several lymphocyte functions. Stress is known to be involved in tumorigenesis or tumor development by the inactivation of cytotoxic T cells and natural killer (NK) cells [34]. In surveys after natural disasters such as earthquakes and hurricanes, associations between disaster-related stress and decrease of immune functions, especially suppression of NK cell activity, have been reported [35-37]. A survey of victims of the 1995 Southern Hyogo Prefecture Earthquake in Japan revealed that post-traumatic stress disorder symptoms and poor lifestyle are associated with low NK cell activity [37].

Reconstruction of the healthcare system after the Great East Japan Earthquake is significant; however, the progress is uneven among the affected areas [38]. Design and construction of a comprehensive medical system, including lifestyle habit improvements, mental health care, and medical screening after a disaster, are essential for preventing health damage to women. During the sub-acute and chronic phases following the Great East Japan Earthquake, there were significant needs for medical and public health assistance that included infectious disease control and mental health care at evacuation facilities [39]. The provision of health and health facilities is a priority issue in the international guidelines on disaster reduction actions for the 15 years to 2030 adopted by the 3rd United Nations World Conference on Disaster Reduction (WCDRR, 2015) [40]. The present study suggests the importance of prompt reconstruction of healthcare systems and the inclusion of cancer screening in these systems to maintain women's health.

References

[1] Brotherton JML, Bloem PN. Population-based HPV vaccination programmes are safe and effective: 2017 update and the impetus for achieving better global coverage. Best Pract Res Clin Obstet Gynaecol. 2018;47:42-58.

[2] Ikeda S, Ueda Y, Yagi A, Matsuzaki S, Kobayashi E, Kimura T, et al. HPV vaccination in Japan: what is happening in Japan? Expert Rev Vaccines. 2019;18(4):323-325.

[3] Komiyama M, Hasegawa K. Comparison of preventive care for cervical cancer between Japan and western countries: A review. J Pharma Care Health Sys. 2017;4: 185.

[4] Australian Institute of Health and Welfare 2018. Cervical screening in Australia 2018. Cat. no. CAN 111. Canberra: AIHW.

[5] Cancer Registry and Statistics. Cancer Information Service, National Cancer Center, Japan.

[6] Yajima A, Higashiiwai H, Sato A, Watanabe M, Hoshi K, Mori T, et al. Mass population screening for cervical cancer in Miyagi Prefecture. Gynecol Oncol. 1979;8: 131-140.

[7] Sato S, Matsunaga G, Konno R, Yajima A. Mass screening for cancer of the uterine cervix in Miyagi Prefecture, Japan. Effects and problems. Acta Cytol. 1998;42: 299-304.

[8] Ikeda, Maria M., and Miharu Matsumaru. Leadership and social innovation

initiatives at the grassroots during crises. Japan Social Innovation Journal 2.1 2012: 77-81.

[9] Koshimura S, Shuto N. Response to the 2011 Great East Japan Earthquake and Tsunami disaster. Philos Trans A Math Phys Eng Sci. 2015;373 https://doi.org/10.1098/rsta.2014.0373.

[10] El Saghir NS, Soto Pérez de Celis E, Fares JE, Sullivan R. Cancer care for refugees and displaced populations: Middle East conflicts and global natural disasters. Am Soc Clin Oncol Educ Book. 2018; 433-440.

[11] Ozaki A, Nomura S, Leppold C, Tsubokura M, Tanimoto T, Yokota T, et al. Breast cancer patient delay in Fukushima, Japan following the 2011 triple disaster: a long-term retrospective study. BMC Cancer. 2017;17: 423.

[12] Ozaki A, Leppold C, Sawano T, Tsubokura M, Tsukada M, Tanimoto T, et al. Social isolation and cancer management - advanced rectal cancer with patient delay following the 2011 triple disaster in Fukushima, Japan: a case report. J Med Case Rep. 2017;11: 138.

[13] Kanjanvaikoon P. Gerber D. Robison WR. Long term impact of natural disaster on cervical cancer demographics. Gynecol Oncol. 2011;123: 446 (Meeting report).

[14] Liu S, Han J, Xiao D, Ma C, Chen B. A report on the reproductive health of women after the massive 2008 Wenchuan earthquake. Int J Gynaecol Obstet. 2010;108: 161-164.

[15] Li XH, Qin L, Hu H, Luo S, Li L, Fan W, et al. Influence of the Wenchuan earthquake on self-reported irregular menstrual cycles in surviving women. Gynecol Endocrinol. 2011;27: 706-710.

[16] Bloem CM, Miller AC. Disasters and women's health: reflections from the 2010 earthquake in Haiti. Prehosp Disaster Med. 2013;28: 150-154.

[17] Sohrabizadeh S, Tourani PhD S, Khankeh HR. Women and health consequences of natural disasters: Challenge or opportunity? Women Health. 2016;56: 977-993.

[18] Goodman A. In the aftermath of disasters: the impact on women's health. Crit Care Obst Gyne. 2016;2: 6.

[19] Yamagami W, Nagase S, Takahashi F, Ino K, Hachisuga T, Aoki D, Katabuchi H. Clinical statistics of gynecologic cancers in Japan. J Gynecol Oncol. 2017 Mar;28(2):e32.

[20] Hamashima C, Aoki D, Miyagi E, Saito E, Nakayama T, Sagawa M, et al. Japanese Research Group for Development of Cervical Cancer Screening Guidelines. The

Japanese guideline for cervical cancer screening. Jpn J Clin Oncol. 2010;40: 485-502.

[21] Sato K, Oikawa M, Hiwatashi M, Sato M, Oyamada N. Factors relating to the mental health of women who were pregnant at the time of the Great East Japan earthquake: analysis from month 10 to month 48 after the earthquake. Biopsychosoc Med. 2016;10: 22.

[22] Sugawara J, Hoshiai T, Sato K, Tokunaga H, Nishigori H, Arai T, et al. Impact of the Great East Japan Earthquake on regional obstetrical care in Miyagi Prefecture. Prehosp Disaster Med. 2016;31: 255-258.

[23] Nishigori H, Sugawara J, Obara T, Nishigori T, Sato K, Sugiyama T, et al. Surveys of postpartum depression in Miyagi, Japan, after the Great East Japan Earthquake. Arch Womens Ment Health. 2014;17: 579-581.

[24] Higuchi Y, Inui T, Sugiyama S, Wakabayashi K, Kuga N, Hosoi T, et al. Aftermath of the 3.11 Disaster: An analytical evaluation using official statistics. ESRI Discussion Paper Series No.286; Economic and Social Research Institute Cabinet Office, Tokyo, Japan, 2012.

[25] H30 Survey on Prefectural Attitude by the Miyagi prefecture; 2018, March, in

Japanese.

[26] Ueda Y, Sobue T, Morimoto A, Egawa-Takata T, Hashizume C, Kishida H, Okamoto S, Yoshino K, Fujita M, Enomoto T, Tomine Y, Fukuyoshi J, Kimura T. Evaluation of a free-coupon program for cervical cancer screening among the young: a nationally funded program conducted by a local government in Japan. J Epidemiol. 2015;25(1):50-6.

[27] Man RX, Lack DA, Wyatt CE, Murray V. The effect of natural disasters on cancer care: a systematic review. Lancet Oncol. 2018;19: e482-e499.

[28] Yokoyama Y, Otsuka K, Kawakami N, Kobayashi S, Ogawa A, Tannno K, et al. Mental health and related factors after the Great East Japan earthquake and tsunami. PLoS One. 2014;9: e102497.

[29] Arnberg FK, Bergh Johannesson K, Michel PO. Prevalence and duration of PTSD in survivors 6 years after a natural disaster. J Anxiety Disord. 2013;27: 347-352.

[30] Leiva-Bianchi MC, Araneda AC. Validation of the Davidson Trauma Scale in its original and a new shorter version in people exposed to the F-27 earthquake in Chile. Eur J Psychotraumatol. 2013;4.

[31] Becker SM. Psychosocial care for women survivors of the tsunami disaster in India.

Am J Public Health. 2009;99: 654-658.

[32] Fernando GA. Assessing mental health and psychosocial status in communities exposed to traumatic events: Sri Lanka as an example. Am J Orthopsychiatry. 2008;78: 229-239.

[33] Orui M, Harada S, Hayashi M. Disaster mental health team of the Sendai City Mental Health and Welfare Center. Practical report on long-term disaster mental health services following the Great East Japan Earthquake: Psychological and social background of evacuees in Sendai City in the mid- to long-term post-disaster period. Disaster Med Public Health Prep. 2017;11: 439-450.

[34] Reiche EM, Nunes SO, Morimoto HK. Stress, depression, the immune system, and cancer. Lancet Oncol. 2004;5: 617-625.

[35] Ironson G, Wynings C, Schneiderman N, Baum A, Rodriguez M, Greenwood D, et al. Posttraumatic stress symptoms, intrusive thoughts, loss, and immune function after Hurricane Andrew. Psychosom Med. 1997;59: 128-141.

[36] Segerstrom SC, Solomon GF, Kemeny ME, Fahey JL. Relationship of worry to immune sequelae of the Northridge earthquake. J Behav Med. 1998;21: 433-450.

[37] Inoue-Sakurai C, Maruyama S, Morimoto K. Posttraumatic stress and lifestyles are

associated with natural killer cell activity in victims of the Hanshin-Awaji earthquake in Japan. Prev Med. 2000;31: 467-473.

[38] Matsumoto K, Yoshikawa H. Human papillomavirus infection and the risk of cervical cancer in Japan. J Obstet Gynaecol Res. 2013;39: 7-17.

[39] Nohara M. Impact of the Great East Japan Earthquake and tsunami on health, medical care and public health systems in Iwate Prefecture, Japan, 2011. Western Pac Surveill Response J. 2012;2(4):24-30.

[40] Aitsi-Selmi A, Egawa S, Sasaki H, Wannous C, Murray V. The Sendai Framework for Disaster Risk Reduction: Renewing the Global Commitment to People’s Resilience, Health, and Well-being. Int J Disaster Risk Sci. 2015;6:164–176

Supporting information

S1 Table. Areas where cervical cancer screening is covered by the Miyagi Cancer Association.

S1 Figure. Change in the cervical cancer screening rate (CCS-R) in 23 areas that were screened at the hospital of a medical center in Miyagi Prefecture. The bar graph shows the CCS-R (left vertical axis; unit, %), and the line graph shows the population (right vertical axis; unit, number of people).

To Reviewer #1:

We appreciate your comments regarding our manuscript entitled "Cervical cancer screening rates before and after the Great East Japan Earthquake in Miyagi prefecture, Japan" (manuscript ID: PONE-D-19-25407). We have revised the manuscript according to your comments and provided detailed responses to the specific comments below.

Maybe have an editor copy edit for grammar.

This manuscript has been strictly edited.

Abstract: say earthquake was a magnitude 9.0 to show the seriousness of the disaster.

We have added the above to the abstract accordingly (lines 22-23).

Intro: authors say screening rates are low in Japan compared to other countries. What are the prevalence rates of cervical cancer in Japan compared to other countries? What about HPV vaccine rates?

We appreciate your comment. We believe it is important to provide information on cervical cancer prevalence and vaccination rates in Japan. We have summarized that information in the Introduction section (lines 36-44; 46-47; 49-50).

Why was it called the 3.11 earthquake?

The abbreviation "3.11" has been used mainly by Japanese mass media since the earthquake occurred on March 11, 2011. We described the above in the Introduction section (lines 57-59). In addition, "3.11" is widely recognized in Japan; however, there may be many readers who are not familiar with this term. Therefore, we have not used "3.11" as an abbreviation in the manuscript.

What is the evidence to support this line: However, the people affected by the disaster are 61 still unstable, and cannot afford to take care of their health.

Several studies have reported that the mental and financial instability of survivors creates "patients' delay" (lines 70-71). We have added references to clarify this and described it in the next paragraph in the revised manuscript (lines 72-82).

Why study cervical cancer and not the other women's health conditions? Not well justified

We have summarized this information in the Introduction and have described it in the Discussion section. We clarified why we targeted a disaster and its impact on cervical cancer screening in this research (lines 72-86).

Table 1 is not useful to a reader unfamiliar with the region. A map would be more useful.

The code numbers in Table 1, which is S1 Table in the revised version, match those in Figure 1B in order to distinguish the areas on the map. It is clearly described in each legend in the S1 Table and Figure 1B in the revised manuscript.

Methods: Is the Miyagi Cancer Society a reputable resource? How do they get their data? Is it a government agency?

The Miyagi Cancer Society was the first established organization in Japan for cancer since 1958. In 2012, the society was recognized by the administrative agency as a public interest incorporated foundation and has undertaken a cancer screening and cancer registration business in Miyagi Prefecture, Japan. The Annual Report is also compiled as one of its functions. We have summarized these details in the Materials and Methods (lines 100-103).

Results: Why only mobile van data included? Seems like a significant limitation. Where do most people get cervical cancer screenings in this region?

Medical exam statics at hospitals or the medical center in Miyagi Prefecture include a large number of occasional outpatients, who complain of symptoms such as irregular bleeding. On the other hand, women who have undergone cervical cancer screening in a mobile van were all periodic health care check-ups. In addition, in these regions, cervical cancer screening is covered by the mobile van. We added above sentences in the Materials and Methods and Results (lines 117-122).

Better visualizations would make the data more meaningful (a map rather than a long table, perhaps). AH OK, I see maps at the end. I like the figures. But better to label the map with the region name and the bar charts with region names. The "T-3" labels are hard to follow.

We agree with your comment. We revised Figure 1B accordingly.

Discussion: what were the results from the pregnancy studies? I still don't know why cervical cancer was the focus here. No solid health or financial justification has been provided. OK page 19-21 are good. Maybe more of this justification or background should be in the introduction.

We agree with your suggestion. We have included a summary of this discussion in the introduction and have clarified our motivations for studying a disaster and its impact on cervical cancer screening (lines 72-86).

What was the population decline?

It is well known that the tsunami resulted in severe human and residential damage as a result of the Great East Japan Earthquake. Therefore, coastal populations are considered to have decreased both due to death as well as migration as a consequence of the tsunami. We added this sentence in the Discussion section (lines 227-230).

Overall it's a decent paper. Though I am still unclear if the magnitude of change in screening is clinically meaningful. Can that point be proven? For example, saying that in Katrina the mean time to diagnosis was 7 years compared to 4 before Katrina – that is clinically meaningful. Could something similar be said here?

We appreciate your kind comments regarding our research. Compared to the USA CCS-R, the Japan CCS-R is too low. Therefore, it would be difficult to perform the same analysis as that in US research due to the small number of comparative patients. Otherwise, in Japan, cervical cancer screening is expected to reduce cervical cancer deaths (recommendation grade B), with evidence levels of 2++ for conventional and 2+ for liquid-based cytology. There are limitations to conducting a further detailed analysis in the areas examined in this study; however, a reduction in CCS-Rs suggests that women's health will be significantly affected. We have clarified this aspect in the Discussion section (lines 274-286).

To Reviewer #2

We appreciate your comments regarding our manuscript entitled “Cervical cancer screening rates before and after the Great East Japan Earthquake in Miyagi prefecture, Japan” (manuscript ID: PONE-D-19-25407). We have revised the manuscript according to your comments and provided detailed responses to specific comments below.

It may be more universally understandable if the magnitude of the earthquake is given in the abstract, i.e., after the Great East Japan Earthquake to put in the parentheses (magnitude 11.0); if the authors decide to leave 3.11 earthquake in the parentheses, they should put it in quotations "3.11" or indicate in some other way that this is a colloquial, synonymous term. The authors do explain this in line 53, however (but the abstract is often the first paragraph read in a manuscript). Elsewhere in the manuscript (line 53, 59), it may be more clear if the authors refer to the earthquake as the Great East Japan Earthquake instead of the 3.11 earthquake.

We agree with your suggestions. In the abstract, “3.11” has not been used as an abbreviation for the Great East Japan Earthquake. In the text, “3.11” was only mentioned as being used mainly by the Japanese mass media and not used as an abbreviation elsewhere (lines 57-59).

In the abstract, the sentence "It is possible that a decrease in CCS-R will lead to the detection in the advanced stages of cancer." may be incorrect? Did the authors want to suggest that a decrease in CCS-R may lead to less detection of advanced stages of cancer?

We apologize for any inaccuracies. As you pointed out, a decrease in the CCS-R may lead to low detection of advanced stages of cancer. We have revised the abstract accordingly (lines 31-32).

In the first sentence of the introduction (line 36), please state why this recommendation is important (otherwise this sentence seems abrupt as an opening to this manuscript).

We agree with your comments. It is important to consider that in the results of this study, cervical cancer screening is recommended based on the evidence from Japan. Therefore, we moved this sentence from the Introduction to the Discussion section in the revised manuscript.

The introduction does not flow well in terms of the verbage used. Lines 41-45 should flow and lead into each other.

We agree with your comments. We have revised this series of sentences in the revised manuscript (lines 36-55).

Lines 55-57 use past and present tense terms. Instead of "may need 10 years for reconstruction" perhaps the authors may consider using "the tsunami-hit area was predicted to necessitate 10 years for reconstruction."

We agree with your comments. We have revised this sentence accordingly (lines 62-63).

Please give a time course in lines 57-61. How long after the earthquake did the mobile van service resume screening?

We agree with your comments. In many areas, cervical cancer screening was resumed in April of the year of the disaster. However, in coastal areas (L-2, L-3, T-3, U-1b in Fig. 1), the re-initiation of screenings was delayed from July to December of that same year. The screening was resumed only in Oshika (L-7 in Fig. 1) in February following the earthquake. We added this information to the Introduction section (lines 66-70).

Line 62: please cite this publication within the manuscript using a standardized method such as MLA format (i.e., "In a study describing survey results regarding reproductive health after the 2008 Wenchuan earthquake in China, Liu et al. reported......")

We agree with your comments. In the revised manuscript, we modified the Introduction section to include this sentence (lines 72-86).

Lines 69-71 is too nebulous and general of a statement to make. The authors should add in that the failure of health management in the specific context of natural disasters (or whatever they feel it is specifically pertaining to).

We agree with your comments. In the revised manuscript, we modified the Introduction section accordingly (lines 72-86).

Line 72: "Japan fiscal year (FY; April 1 to March 31) 2009–FY2016" should be simplified to

set years (i.e., 2009-2016).

We agree with your comments. We have revised the manuscript accordingly (line 87).

Table 1 is too complex and slightly unnecessary. Perhaps a small color coded index included as a legend in Figure 1 with the names of the prefectures would suffice (with consideration to take out Table 1 entirely; the prefectures can remain in Table 2 since it also provides pertinent information).

We agree that Table 1 may be unnecessary for many readers. However, it may be helpful for readers who know Japan in order to identify a location. We have left it as a supplemental table. In addition, we have revised Figure 1 to make it easier to understand.

Line 93: instead of "people," should this be "females"? Why is the CCS-R pertaining to those 20 years old and over - if this is a screening protocol in Japan then please state that here.

We appreciate your polite comment. Line 93 "people" has been corrected to "women".

The incidence of cervical cancer has increased in Japanese women in their mid-20s. Therefore, Japanese cervical cancer screening guidelines recommend to begin screening at the age of 20 years. We described this in the Materials and Methods section (lines 105-107).

It would be useful to get an idea about the numbers affected since -3.0% etc...(e.g., line 113, line 121) may be difficult to interpret meaningfully to the reader. The authors should consider detailing the population numbers in the text and delineating the axes on the graphs in all of the figures themselves (including the supplementary ones) for whatever they represent (i.e., year, population in thousands etc...) instead of in the figure legend.

We agree with your comments. We have added new graphs in Figures 2 and 3 to make it easier to understand the decrease in cervical cancer screening rates. We also revised the graphs accordingly.

The authors may consider not stating: "no significant difference was found" in line 170 since the p value was 0.6303. Likewise, in line 180 it is stated that there was a significant decrease in the coastal area compared with that in other areas however this is difficult to interpret without a regression analysis (and consideration of determining a p value).

We apologize for failing to explain Figure 5. We divided the regions into coastal and

non-coastal areas and compared them using the Mann–Whitney U test. The *p*-value shows the results. We have added the legend for Figure 5 (lines 211-215)..

Lines 196-200 are unclear in their message?

In this article, it was suggested that the victims may be motivated to manage their health if they realize that there is recovery from disaster. However, there is no evidence or reference to support this suggestion as you point out. I agree that this is unclear. Therefore, we have deleted this text from the revised manuscript.

Lines 204-206: even though the authors could not identify causes, are there any that could be hypothesized based on data from previous similar studies?

We agree with your comments. In an evidence review by CPSTF (USA), small media sources such as newspapers and educational videos as well as solicitation by telephone or letter are recommended as interventions to increase the CCS-R (https://www.thecommunityguide.org/content/task-force-findings-cancer-prevention-and-control; access 2020.1.17). In Japan, it was also reported that local government initiatives and education contributed to increasing the screening rate of young women. However, a detailed investigation is required in areas where these screening rates have declined and awareness attempts by local government and other organizations may have been delayed. We have added this to the Discussion section (lines 248-257).

I was not able to easily find the information for reference #23. Is New Orlando, USA a city that was described in this study?

Ref.#23 is an abstract of the 2001 Western Association of Gynecologic Oncologists (WAGO) Annual Meeting published in Gynecologic Oncology. We noted this in the manuscript that this reference is a conference abstract. The DOI number is also added to link to this abstract (lines 78-80).

Line 218: what stage is being referred to? Lines 219-221 should be considered being combined.

The details of the stage data are described in the Discussion section. We also revised the manuscript as you pointed out (lines 270-273).

Why is this important for clinical outcomes? Is there any data regarding how many cervical

cancers were missed as a result of delayed screening (either from the authors data set or from any of the references)?

The five-year overall survival rates for cervical cancer stages I and II are known to be approximately 90% and 75%, respectively. Early detection by periodic screening is critical in order to prevent death from cervical cancer. If regular screening is skipped as a result of the earthquake, it may be detected later as advanced cancer. This condition was found in the area that was impacted by Hurricane Katrina.We added this in the Discussion section (lines 273-282).

Lines 235-236 contain repeated information which was just mentioned in lines 232-234.

We deleted this sentence in the revised manuscript.

Lines 239-242 need to be written with a more clear introduction or transition. Lines 241-242 are unclear.

It is known that smoking increases the risk of HPV infection. In this sentence, it suggests the number of smokers is increasing in many affected areas, implying that cervical cancer should be considered in these areas. However, there is no direct evidence of smoking rates in coastal and non-coastal areas surveyed in this research. Therefore, we removed this information from the revised manuscript.

Consider writing a separate conclusion since lines 244-248 do not transition in a clear way since the discussion prior to it pertains to smoking a mental stress.

We removed the description of mental stress and smoking and added a separate conclusion in the revised manuscript.

The conclusive remark (line 246-248) is meaningful however there is lack of data to support this claim. The authors should consider including evidence from the literature supporting why design and construction of a comprehensive medical system etc... would be beneficial.

During the sub-acute and chronic phases following the Great East Japan Earthquake, there were significant needs for medical and public health assistance that included infectious disease control and mental health care at evacuation facilities. The provision of health and health facilities is a priority issue in the international guidelines on disaster reduction actions for the 15 years to 2030 adopted by the 3rd United Nations World Conference on Disaster Reduction (WCDRR, 2015). The present study suggests the importance of prompt

reconstruction of healthcare systems and the inclusion of cancer screening in these systems to maintain women's health. We have described this in the Discussion section as a conclusion (lines 306-314).

To Reviewer #3:

We appreciate your comments regarding our manuscript entitled “Cervical cancer screening rates before and after the Great East Japan Earthquake in Miyagi prefecture, Japan” (manuscript ID: PONE-D-19-25407). We have revised the manuscript according to your comments and provided detailed responses to specific comments below.

The study utilized appropriate statistics in analyzing data collected from relevant sources. The conclusions drawn and recommendations made were based on the results of the study. However, the following observations and comments addressed.

1. The cervical cancer screening rate for Japan should be stated for adequate comparison with other countries (lines 40-41).

We have added cervical cancer screening rates for Japan (33.7%; age group: 20-69 years) and Miyagi Prefecture (42.1%; age group: 20-69 years) (lines 46-50).

2. Recast the sentence on lines 50 - 51 removing 'screening for cervical cancer screening' so the sentence reads 'All mobile van screenings are population-based'.

We appreciate your comments. We have revised this sentence accordingly.

3. Replace 'as per' with a standard English word or phrase (line 65).

This phrase was considered meaningless and has been removed from the revised manuscript.

4. Replace 'i.e' with the appropriate words (line 97).

This word has been corrected to "*e.g.*" (line 113).

5. Lines 196 - 197: Report in the past.

We appreciate your comment. This sentence was deleted due to the other reviewers' suggestions.

References.

6. Cross-check if the following articles are single-paged articles: reference nos [3], [14], [21], [39]; and provide the complete pages where missing.

These references are single-paged online journals.

7. Provide the year of publication for no [5], and page numbers for no [27]. Also check the correctness of the page number for reference no [38].

Reference No. [5] was 2016 public data of National Cancer Center in Japan. Nos. [27] and [28] are also online journals, each having a single number "4" and "e018943".
