# Supplementary material for: Cervical cancer screening rates before and after the Great East Japan Earthquake in the Miyagi Prefecture, Japan
Source: PLoS One. 2020 Mar 11;15(3):e0229924. doi: 10.1371/journal.pone.0229924 (PMC7065810; doi:10.1371/journal.pone.0229924)
Supplement: Attachment — Submitted filename: To_reviewer#3.docx [file pone.0229924.s005.docx]

To Reviewer #3:

We appreciate your comments regarding our manuscript entitled “Cervical cancer screening rates before and after the Great East Japan Earthquake in Miyagi prefecture, Japan” (manuscript ID: PONE-D-19-25407R1). We have revised the manuscript according to your comments and provided detailed responses to specific comments below.

***Authors should make the following corrections:***

***Lines 39 - 40; the data source should be appropriately referenced by including it in the reference list and represented with the reference number in block parenthesis in the text.***

We agree with your comments. We have revised manuscript accordingly (line 39 and reference #1).

***Line 113; the appropriate phrase for i.e, is 'that is', not e.g.***

We appreciate your comments. We have corrected manuscript accordingly (line 112).
